# Genetic basis and role of exotic accessions in cultivated cotton fiber quality improvement

**DOI:** 10.1007/s00122-025-05043-2

**Published:** 2025-09-27

**Authors:** Ankush Sharma, Mingrui Xu, Deepak Vitrakoti, Jinesh D. Patel, Peng W. Chee, Andrew H. Paterson

**Affiliations:** 1 Plant Genome Mapping Laboratory, University of Georgia, Athens, GA 30602 USA; 2 Department of Crop, Soil and Environmental Sciences, Auburn University, Auburn, AL 36849 USA; 3 Department of Crop and Soil Sciences, The University of Georgia, Tifton, GA USA; 4 NESPAL Molecular Cotton Breeding Laboratory, The University of Georgia, Tifton, GA USA

## Abstract

**Key message:**

Exotic Gossypium accessions still harbor QTL‑validated alleles that, combined with CRISPR pyramiding and genomic selection, can break the entrenched fiber length–strength trade‑off.

**Abstract:**

Cotton’s four independent domestications twice in diploids and twice in allotetraploids offer a natural experiment in fiber improvement. Synthesizing three decades of data, we chart how polyploidy, selection and modern breeding have repeatedly reshaped the *Gossypium* genome. More than 15,000 quantitative trait locus (QTL) and genome wide association mapping studies (GWAS) hits converge on a handful of chromosomal “hotspots”; new MAGIC, NAM, NIL and long-read resources now narrow these peaks to < 200 kb, resolving causal genes such as *GhHOX3, GhZF14* and *GhMYB7*. Multi-omics evidence links auxin, ethylene, gibberellin, brassinosteroid and strigolactone signaling to HDZIP IV, MYB, bHLH/HLH and ERF networks that drive fiber initiation, extreme cell elongation and cellulose deposition. Population genomics shows that ~ 40% of favorable fiber alleles are fixed in elite *Gossypium hirsutum*, yet wild diploids and landraces still harbor variants that could break the length strength trade-off. We propose a three-step roadmap genomic selection, CRISPR gene pyramiding and accelerated introgression to expand cotton’s genetic base and deliver fibers suited to sustainable textile demands.

**Supplementary Information:**

The online version contains supplementary material available at 10.1007/s00122-025-05043-2.

## Introduction

Cotton (*Gossypium* spp.) is unrivaled among crop models for studying parallel domestication. At least four independent selective events twice in diploids (*Gossypium  arboreum*, *Gossypium  herbaceum*) and twice in allotetraploids (*Gossypium  hirsutum*, *Gossypium barbadense*) arose in Mesoamerica, coastal South America, the Indian subcontinent and Northeast Africa/Arabia (Cedroni et al. 2003; Doebley et al. 2006; Grover et al. 2022; Viot & Wendel 2023). Collectively they provide a replicated natural experiment for dissecting convergent evolution of complex traits under contrasting ploidy states (Yoo & Wendel 2014) (Olsen & Wendel 2013a, b).

Eight diploid genome groups (A–G, K) anchor the genus on three continents (Craven et al. 1994; Fryxell 1992). Hybridization between ancestral A- and D-genome species during the mi Pleistocene produced an allotetraploid progenitor whose descendants dominate modern agriculture. Classic selection experiments on natural allopolyploids first showed that allopolyploidy releases novel, heritable variation beyond the parental range (Jiang et al. 1998); genomic surveys now reveal the underlying cis- and trans-regulatory rewiring.

Domestication reshaped both plant architecture and fiber morphology, converting short, coarse, pigmented seed hairs into the long, fine, white lint demanded by industry (Grover et al. 2022; Li et al. 2005). Fiber quality is quantitative, encompassing length, strength, fineness, uniformity and elongation (Arpat et al. 2004; Chaudhary et al. 2008; Hovav et al. 2008). Quantitative trait loci (QTL) mapping, genome wide association mapping studies (GWAS) and transcriptome-guided candidate cloning have uncovered hundreds of loci and several causal genes including *GhHOX3*, *GhMYB7* and *GhZF14* that govern fiber initiation, 30,000-fold cell elongation and secondary wall cellulose deposition (Jiang et al. 1998; Abdurakhmonov et al. 2008; Hinchliffe et al. 2011; Lacape et al. 2005; Liu et al. 2018; Mei et al. 2004; Rapp et al. 2010; Shi et al. 2006; Yoo & Wendel 2014)) have collectively provided valuable insights into the genetic architecture of fiber development.

While these efforts have advanced functional knowledge, intensive selection has narrowed diversity within the elite upland gene pool (Chee et al. 2004; May et al. 1995). SSR- and SNP-based diversity studies reveal extensive haplotype fixation and show that a significant portion of favorable fiber alleles are already near fixation in elite cultivars (Van Esbroeck et al. 1998; Zhu et al. 2019). Conversely, wild diploids, landraces and rare island tetraploids retain alleles that could break the entrenched length versus strength trade-off or confer stress tolerance (Chen et al. 2007; Li et al. 2014; Ma et al. 2021; Wang et al. 2019).

Technology is closing the genotype–phenotype gap. High-density SNP arrays, pangenomes and long read assemblies delineate sub-200 kb QTL intervals (Blenda et al. 2012; Udall et al. 2006) (Supplementary Table 1). High-throughput phenotyping platforms offer complementary precision: cost-efficient high-volume instrument (HVI) systems deliver indirect proxies, whereas advanced fiber information system (AFIS) provides direct measures of fineness and maturity at lower throughput (Hequet et al. 2006; Paudel et al. 2013). Integrated omics studies now map hormone signaling (auxin, ethylene, GA, BR, strigolactone) onto HDZIP IV, MYB, bHLH-/HLH and ERF transcriptional networks that orchestrate each developmental stage (Qin et al. 2007; Qin & Zhu 2011; Shan et al. 2014; Walford et al. 2011, 2012; Xiao et al. 2019). Proteomic and metabolomic profiling further highlight the roles of ROS homeostasis and cell wall enzymes in modulating cellulose biosynthesis (Carpita 2011; Lerouxel et al. 2006; Miller et al. 2010; Mittler et al. 2004; Pradeep Reddy et al. 2002; Xu et al. 2007).

Despite this progress, yield–quality antagonism remains a central breeding hurdle (Bednarz et al. 2005; Bradow & Davidonis 2000). Modern, integrated frameworks that combine multi-environment QTL analyses with RNA-seq-guided candidate prioritization and genomic prediction are improving cross-environment accuracy and helping pinpoint stable, pleiotropy-aware targets (Abdalla et al. 2001; Gulati & Turner 1929; Wendel & Grover 2015). In parallel, Clustered Regularly Interspaced Short Palindromic Repeats (CRISPR) allele editing and gene-stacking strategies have begun to validate causal loci and provide practical routes to pyramid favorable haplotypes while minimizing linkage drag (Du et al. 2018; Ma et al. 2018; Zhang et al. 2015). Equally important is the systematic mobilization of crop wild relatives now accelerated by speed breeding, advanced backcrossing and genomic selection to broaden allelic diversity for yield stability under variable environments (Renzi et al. 2022; Varshney et al. 2021). Community resources and dense marker platforms further shorten the path from discovery to deployment, enabling faster iteration from QTL to candidate gene to validated allele in breeding pipelines.

**Fig. 1:**
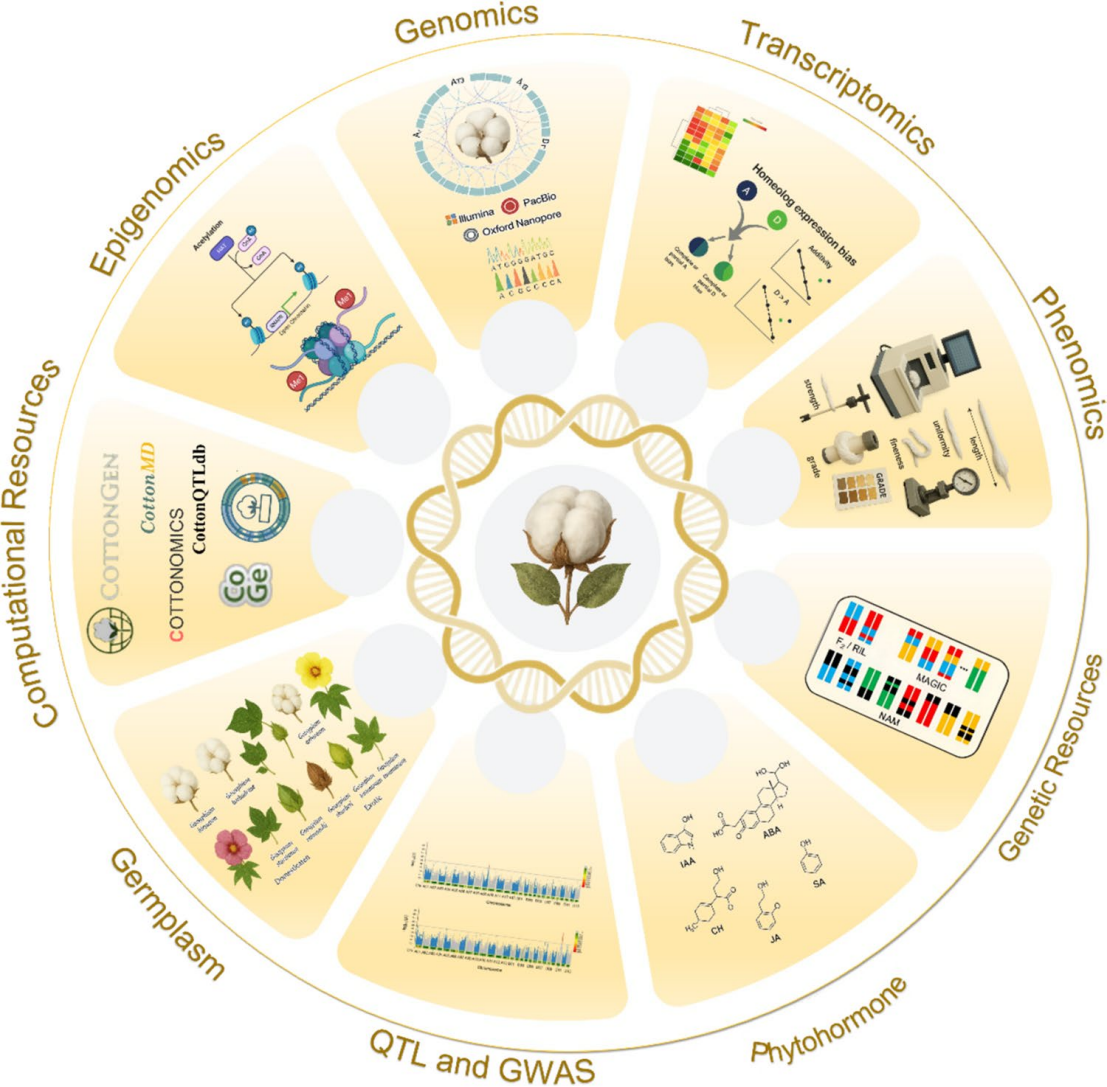
Integrated Strategies in Cotton Research

In this review, we integrate three decades of genetics, genomics and phenomics to create a comprehensive picture of cotton fiber biology. First, we juxtapose modern cultivars with wild relatives and traditional landraces to expose reservoirs of untapped diversity, illustrating how domestication and polyploidy have channeled but not exhausted the genetic potential for fiber improvement. Second, we assemble a global catalog of all published QTL and GWAS signals for fiber length, strength, fineness, uniformity and elongation, providing the most exhaustive map of trait associated loci to date. Third, we synthesize candidate gene discoveries spanning both diploid and allotetraploid backgrounds and weave them into a stage-specific regulatory network that unites hormone signaling with HDZIP IV, MYB, bHLH-/HLH and ERF modules. Finally, we outline a tiered roadmap that moves from hotspot anchored genomic selection, through CRISPR-based allele stacking, to accelerated introgression and speed breeding, charting practical routes toward fibers that satisfy the dual challenges of climate resilience and market demand (Fig. 1).

Clockwise from top: (1) reference genomes and population resequencing; (2) transcriptomics that tracks homeolog bias and stress responses; (3) high throughput phenomics across environments; (4) structured populations (NAM, MAGIC, RILs) for fine mapping; (5) phytohormone profiling of fiber growth and stress adaptation; (6) QTL + GWAS linking loci to fiber and yield traits; (7) germplasm mining of wild, landrace and elite accessions; (8) bigdata infrastructure (e.g., CottonGen); and (9) epigenomics of chromatin and DNA methylation. Together these modules create a seamless discovery-to-deployment engine for superior fiber alleles.

## Diversity in wild versus domesticated cotton fibers

Domestication converted cotton’s short, pigmented seed hairs into the long, white lint that underpins the modern textile industry, yet elite breeding has captured only a fraction of the genetic solutions scattered across *Gossypium*. Wild *G. hirsutum* (Yucatán/Caribbean) and *G. barbadense* (Peru–Ecuador, Galápagos) still spin fibers < 15 mm with high micronaire, but harbor rare alleles that decouple fineness from yield or add stress resilience (Applequist et al. 2001; Wendel et al. 1992). A striking recent example is a single-base deletion in *GhROPGEF5*: introgressing the favorable allele adds ~ 1.5 mm length and ~ 1.8 g tex⁻^1^ strength without penalizing yield (Wang et al. 2025).

**Fig. 2:**
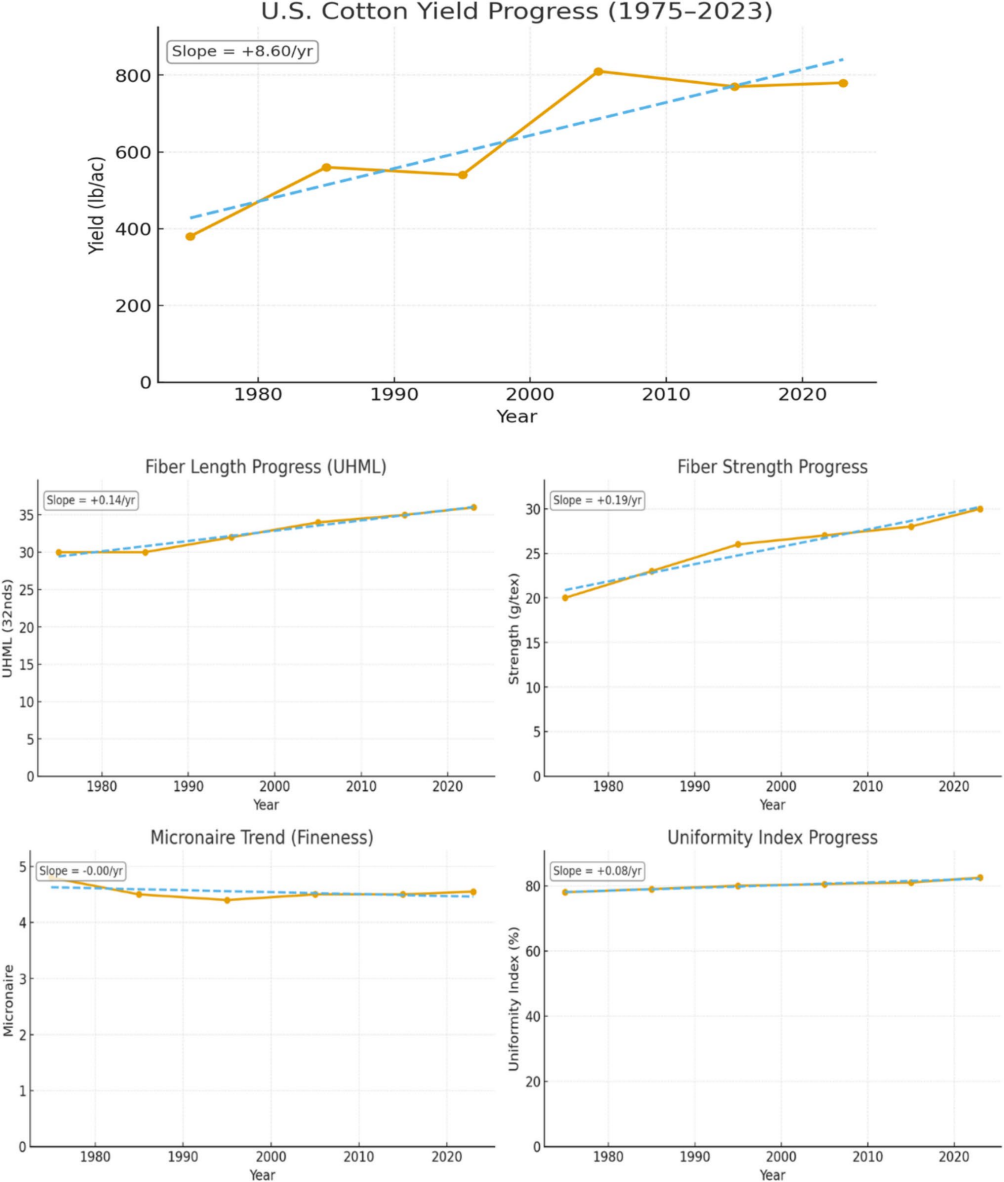
Trends in US cotton yield versus fiber‐quality traits (1975–2023)

Yield advances have outpaced quality. US lint output doubled between 1975 and 2023 (+ 8.6 lb acre⁻^1^ yr⁻^1^), whereas upper half mean length and tensile strength edged up only 0.14 32nds and 0.19 g tex⁻^1^ per year; micronaire even drifted upward (Fig. 2; USDA-NASS; Cotton Inc.). HVI classing blurs diameter with maturity, hiding the elusive “thin-and-mature” fibers breeders need (Kelly et al. 2012). The potential solution is a diversity first pipeline: deploy AFIS or single-fiber imaging on exotic rich panels, flag superior haplotypes, then convert each into low-cost SNP/InDel tags so commercial programs can track fine fiber genetics by genotyping, not rephenotyping (Hequet et al. 2006; Paudel et al. 2013).

Old-World diploids follow a parallel path. Truly wild *G. arboreum* is now almost extinct; *G. herbaceum* survives as scattered ferals. Both spin 10–22 mm fibers shorter and weaker than upland or Pima lint (Hu et al. 2019), yet landraces preserve alleles for robustness under heat and drought. Genomic scans (F_ST_ ≈ 0.41) show substantial divergence between the two A-genome crops, but chromosome–scale introgression on A07 and A10 betrays historical gene flow that may already have shared favorable fiber alleles (Abdul Kadir 1976; Gulati & Turner 1928).

National averages (USDA‐NASS yield data; Cotton Incorporated Crop Quality Summaries) show lint yield rising from ~ 380 to ~ 800 lb/acre (+ 8.6 lb acre⁻^1^ yr⁻^1^, ≈ 2.2%/yr), while fiber length (UHML) improved only ~ 0.14 32nds/yr, strength ~ 0.19 g tex⁻^1^/yr and uniformity ~ 0.08 units/yr. These contrasting slopes underscore that, despite steady yield gains, quality traits advance much more slowly. The figure’s dashed trend lines and annotated slopes highlight this disparity. Sources: USDA‐NASS (nass.usda.gov); Cotton Incorporated Crop Quality Summaries (cottoninc.com).

Wild tetraploids widen the palette still further. *G. tomentosum* contributes D-genome haplotypes that simultaneously raise strength and length without yield drag (Hovav et al. 2008; Hu et al. 2019; Zhang et al. 2011), while *G. darwinii* offers variants that extend elongation and control natural pigmentation (Wendel & Percy 1990). A 2025 *G. barbadense* pangenome built from 12 new assemblies pinpoints structural variants underlying length, strength and lint percentage, revealing active allele exchange with upland cotton and providing breeder friendly markers for introgression (Meng et al. 2025). Complementary GWAS on single-fiber traits now map within boll length heterogeneity to a handful of bundle strength loci, sharpening the breeder’s target list (Kim et al. 2024a, b).

Domestication funneled *Gossypium* diversity through a narrow neck. Whole genome surveys show that modern upland cultivars capture < 10% of the variation present in wild *G. hirsutum*, while cultivated *G. barbadense* arose from an equally small founder pool (Iqbal et al. 2001; Wendel et al. 1992). A new pangenome built from 1961 accessions reveals > 70,000 presence/absence variants, most missing from elite lines yet common in coastal “dooryard” races and island wilds (Meng et al. 2025). These semi-cultivated *G. hirsutum* long grown outside formal programs often display intermediate fiber quality and harbor rare alleles lost during modern improvement (Gulati & Turner 1928; Ning et al. 2025). Their conservation and systematic evaluation are now explicit targets of the US National Cotton Germplasm Collection and recent USDA-ARS initiatives (ars.usda.gov).

Phenotypic contrasts between wild and domesticated forms spotlight developmental ceilings. Wild fibers initiate sooner, elongate for a shorter window and are fewer per seed, capping length near 15 mm; yet they can match cultivated lint in tensile strength (Applequist et al. 2001). Domestication therefore seems to have prioritized length gains while leaving strength alleles near their natural maximum. Old-World diploids tell a complementary story: despite overall short staple, some *G. arboreum* landraces combine surprising strength with drought hardiness, traits likely favored under traditional rain-fed systems (Iqbal et al. 2015; Mehetre et al. 2003). Genomic scans detect chromosome-scale introgression between *G. arboreum* and *G. herbaceum*, hinting that farmer-mediated hybridization already shuffled favorable fiber alleles across species boundaries.

Altogether, these findings confirm that each domestication followed its own genetic trajectory yet converged on spinnable, uniform lint. Wild, feral and landrace populations remain a reservoir of untapped alleles now visible through pan-genomics and ripe for precision introgression that can help break the current fiber quality plateau in cotton breeding.

## Mapping fiber quality: QTL, GWAS and beyond

The first linkage maps built from simple-sequence repeats showed that every measurable lint attribute length, strength, fineness, elongation, color was genetically complex (McCarty Jr. et al., 1996). This insight was foreshadowed by the pioneering SSR map (Kohel et al. 2001) and the first high-density SNP map (Yu et al. 2012), both of which revealed hundreds of loci per trait across the A and D subgenomes. Work in elite × wild or *G. hirsutum* race populations revealed favorable alleles entirely absent from modern cultivars, confirming that early domestication had already fixed many “big” fiber genes while leaving smaller-effect variants in landraces and diploids (Alkuddsi et al. 2013). At the same time, quantitative trait locus (QTL) screens in classical RILs rapidly multiplied: by 2002 Paterson’s group had cataloged six length QTL in a single F₂ and shown that irrigation or heat stress could flip both effect size and significance, foreshadowing the central role of environment in cotton genetics. Their genotype-by–environment analysis remains a milestone for fiber G × E studies (Paterson et al. 2003). (Table 1).

**Table 1:** Representative QTL and GWAS loci associated with cotton fiber quality traits

Trait	Chromosome	Population/study	Notable finding	Number of QTLs	Source
Fiber length (FL)	A3 (Chr 3)	Meta-QTL studies	QTL cluster identified in ≥ 5 datasets; consistent positive allele effects	60	(Lacape et al. 2010)
Fiber length (FL)	D11 (Chr 21)	MAGIC (11-parent upland cotton)	GWAS locus at ~ 24.6 Mb; candidate gene *GhZF14* (MATE transporter) with nonsynonymous SNPs influencing fiber length	9	(Wang et al., 2023b)
Fiber length (FL)	A-genome (multiple)	*G. arboretum* GWAS (215 accessions)	177 significant SNPs; 56 candidate genes (e.g., glycosyltransferases, TFs) identified via expression QTL analysis		(Iqbal et al. 2021)
Fiber length (FL)	Linkage group A03	*G. hirsutum* cv Siv’on × *G. barbadense* cv F-177 900 interspecific F_2_ Population	A total of six fiber length QTLs were detected with statistical significance in one or more data sets. A QTL at *LG A03* explained13.7% of phenotypic variance (large effect)	24	
Fiber length (FL)	D9 (Chr 23)	A total of 99 *Gossypium hirsutum* L. and two *Gossypium barbadense* L. germplasm accessions	A marker loci *TMO06* at ~ 109.24 M explained 16.86% to 19.56% of phenotypic variance across 2 years (large effect)	30	(Cai et al. 2014)
Fiber length (FL)	D1 (Chr 15), D6 (Chr 25)	TM-1 × NM24016 recombinant inbred mapping population	Two QTLs were detected for length uniformity, explaining 22.3% (D1) and 14.33% (D6) of the phenotypic variance	6	(Gore et al. 2014)
Fiber length (FL)	A13 (Chr 13)	MAGIC population	Using AFIS single-fiber measurement method identified *qFL-A13-1* associated with the number-based length might be a potential candidate to be used for improving fiber length without affecting the weight-related property	5 for weight-based AFIS Lw, 5 for AFIS UQLw	(Kim et al. 2024a, b)
Fiber strength (FS)		Three RILs from 7235 × TM-1	A QTL, *qFS-F*_*2:3*_*-BES-3*, explained phenotypic variance of 14.8%. The CI of *qFS-3* was 0.11–0.66 cM from 3 to 4 SSR markers	22	(Chen et al. 2009)
Fiber strength (FS)	A02 (Chr 2)	BC_3_F_2_(TX)(GA)	A QTL, qFS02.112.9% to 13.1% variance explained	4	(Zhang et al. 2011)
Fiber strength (FS)	A06 (Chr 6)	F_2_/F_2:3_ populations and RIL population	A QTL, *qFS*_*A06*_ (90.74 Mb –90.83 Mb), explained 5.10% of the phenotypic variance	4	(Tang et al. 2024)
Fiber elongation (ELO)	A03 (Chr 3)	Two new F_2_ populations derived from crosses between *G. barbadense* var. Pima S-7 and *G. hirsutum* cv. Texas Marker-1 (TM-1) isogenic lines with *n2* and *im* mutants	A QTL, *EL03.1* (71 Mb -102.7 Mb), explained 23.9% variance explained (large effect)	33	(Rong et al. 2007)
Fiber elongation (ELO)	D08 (Chr 24)	Upland cotton “Xinza 1” (*G. hirsutum*) from the cross of GX1135 (*G. hirsutum*) (P1) and GX100-2 (*G. hirsutum*) (P2). A population of 173 F_2:3_ family lines	A large effect and stable QTL, *qFE-chr24-1*, explained 10.76% of the phenotypic variance	11	(Liang et al. 2013)
Fiber elongation (ELO)	D01 (Chr 15)	F_2_, F_2:3_ and recombinant inbred lines (RILs, F_6:8_) populations	QTL *qFE-C15-1* ~ 12.86% variance explained in F2 population and ~ 7.7% variance explained in F2:3 population.QTL *qFE-C7-1* ~ 11.53% variance explained in RILs,F_6:8_ population	5	(Sun et al., 2024)
Fiber elongation (ELO)	D11 (Chr 21)	BC1 population of chromosome segment substitution line (CSSL) population including 553 individuals was established using *G. darwinii* accession 5–7 as the donor parent and *G. hirsutum* cultivar CCRI35 as the recipient parent	QTL *qFED11.1* ~ 2.2% to 4.9% variance explained in three different populations	17	(Wang et al. 2024)
Upper half mean length (UHML)	A02 (Chr 2)	Two new F_2_ populations derived from crosses between *G. barbadense* var. Pima S-7 and *G. hirsutum* cv. Texas Marker-1 (TM-1) isogenic lines with *n2* and *im* mutants	QTL *FLA02.15(HVuhm)* ~ 27.9% variance explained (large effect)	2	(Rong et al. 2007)
Upper half mean length (UHML)	D02 (Chr 14)	BC_1_F_2:3_	The percent phenotypic variance explained (PVE) values for 2 QTL (*qUHM_D02_1*, *qUHM_D02_2*) were about 1.59% across the populations	12	(Mangla et al. 2025)
Upper half mean length (UHML)		BC_3_F_2,_ BC_3_F_2:3,_ BC_3_F_2:4_	QTL *qUHM-24–2* ~ 16.23% to 32.01% variance explained	19	(B. Wang et al. 2017a, b)
Upper half mean length (UHML)	D09 (Chr 23)	3–79 (with long fiber length and high fiber strength) as the paternal parent and E22 (with high yield and strong drought resistance) as the maternal parent. A CSSL population of 319 accessions	QTL q.DRC_FUHML.D09.1; q.BSA.D09.1 identified by three strategies, ~ 7.59% variance explained	8	(Han et al. 2024)
(UHML)	D05 (Chr 19)	264 BC_4_F_5_ lines	QTL *qUHML-D05* could increase the UHML with PVE values from 7.98 to 18.17%	24	(Yang et al. 2023)
Fiber micronaire (Mic)	D06 (Chr 25)	An RIL population of 231 lines was developed from a cross between two homozygous upland cotton cultivars, Lumianyan28(LMY28), Xinluzao24 (XLZ24)	QTL *qFM-chr25-1* and *qFM-chr25-2*, ~ 12.81% and 12.90% variance explained, respectively	52	(Liu et al. 2018)
Fiber micronaire (Mic)	A03 (Chr 3)	250 backcross inbred lines (BILs), developed from an interspecific cross of upland cotton CRI36 × Egyptian cotton (*G. barbadense*) Hai7124	QTL *IqMIC-At3-1* ~ 12.56% variance explained	25	(Pei et al. 2021)
Fiber micronaire (Mic)		249 sea island cotton accessions	Three stable Mic-associated QTLs—*TM4582, TM3628* and *TM82241* were identified with association times greater than 6 in both normal and salt environments	11	(Su et al. 2020)
Fiber micronaire (Mic)	A01 (Chr 1)	269 G. barbadense accessions	QTL *qtl21* ~ 7.3391% variance explained	148	(Song et al. 2024)
Fiber micronaire (Mic)	D03 (Chr 17)	(CCRI35 x Pima S-7) progeny, 600 BC_3_F_2_	One QTL (*qFM-Chr17-2*) was identified in four environments. 2.4%-6.9% variance explained	20	(Yang et al. 2022)
Fiber uniformity index (UI)	A03 (Chr 3), A04 (Chr 4), D04 (Chr 22)	MAGIC population (372 recombinant inbred lines (RILs))	Novel QTLs on chromosome A03 (42 Mb), A04 (93 Mb) and D04 (34 Mb)	3	(Mohammed et al. 2025)
Fiber uniformity index (UI)	D07 (Chr 16)	BC_3_F_1_	A QTL *qUI-16–1*, explained 30.75% of phenotypic variance	8	(Chen et al. 2020)
Short fiber content (SFC)	A01 (Chr 1)	Elite diversity panel (384 upland cotton accessions)	A stable MTA, *SFC_A01* explained 10.82% of phenotypic variance	2	(Gowda et al. 2024)
Short fiber content (SFC)	A07 (Chr 7)	550 Random-mated recombinant inbred population	A QTL *qSFC-c7*, explained 5.21% of phenotypic variance	15	(Fang et al. 2014)

*At* A-subgenome chromosome of tetraploid, *Dt* D-subgenome chromosome, *QTL* quantitative trait locus, *GWAS* genome-wide association study, *IL* introgression line

Lacape et al. (2010) aggregated ~ 600 fiber QTL from independent studies and found striking convergence: chromosome 3 (At3) is a reproducible hub for fiber length, At15 for fineness (micronaire), and Dt6, 8, 25 for natural fiber color. Each region was supported by at least five unrelated experiments, making them trustworthy targets for breeders. Yet the same survey warned that barely half of all published QTL replicated across populations clear evidence that many loci are modest in effect or conditional on background and climate. Rarely, single loci explain double-digit percentages of variance (Rong et al. 2007; Said et al. 2013). Paterson et al. (2003) located a length QTL on A03 (~ 14% PVE) in an Upland × Pima F₂. For uniformity, Gore et al. (2014) detected D1 and D6 QTL explaining 22% and 14%, respectively. Public repositories, such as CottonGen, now host thousands of QTL and GWAS hits; one 2024 review counted ≈1 850 well-curated fiber QTL for length, strength and fineness alone (Baghyalakshmi et al. 2024; Fang et al. 2017; Yu et al. 2013), and the live CottonGen portal lists tens of thousands of marker–trait records spanning 31 distinct fiber traits. This breadth is a double-edged sword: powerful for meta-analysis, yet noisy without rigorous validation.

Resolution leapt forward when an 11-parent MAGIC population (550 RILs) was sequenced. Dual GWAS approaches (SNPG-WAS and identity by descent haplotype GWAS) uncovered 39 fiber loci; two stood out (Fang et al. 2023; Wang et al. 2023b). A D11 QTL, narrowed to a 130-kb window, implicated *Ghir_D11G020400* (*GhZF14*, a MATE transporter) as the causal gene for longer fibers, while an A07 haplotype carrying elevated 20-DPA expression of *Ghir_A07G020390* boosted bundle strength (Fang et al. 2023; Wang et al. 2023b). These examples show how multi-parent designs and transcriptomics can convert anonymous QTL into actionable genes.

Beyond the single MAGIC study, multiple non-MAGIC populations reinforce that cotton fiber quality traits are highly polygenic with mostly small per-locus effects, making them good candidates for genomic prediction. Large GWAS diversity panels, e.g., 719 accessions genotyped with CottonSNP63K (Sun et al., 2024), 276 accessions (Liu et al. 2020), 242 accessions across multiple environments (Zhu/Song et al., 2024), 612 accessions using a 40 K chip (Chen et al., 2020) and an elite US panel of 348 lines tested in 10 environments (Gowda et al. 2024) consistently detect many loci with modest PVE for fiber length, strength and micronaire (Liu et al. 2020). Multi-environment biparental resources similarly show numerous but environment-dependent QTL of modest effect (e.g., RIL/BC designs and high-density linkage maps) (Shang et al. 2016).

Introgression resources, such as the classic chromosome-substitution (CS-B) lines, provide orthogonal validation and transfer of favorable alleles for fiber properties (including improved micronaire in CS-B17 derivatives) (Saha et al. 2006). Trait definition also matters: using AFIS single-fiber metrics, a number-based length measure revealed a distinct A13 locus that was not apparent with conventional HVI/weight-based metrics (Kim et al. 2024a, b), underscoring how refined phenotyping uncovers otherwise hidden loci (Kim et al. 2024a, b). Together with the MAGIC analysis showing widespread epistasis and many small-effect QTL (Wang et al. 2022), these results motivate genomic selection—already demonstrating strong cross-validated accuracies for fiber length/strength and competitive performance for yield in large breeding panels (Li et al., 2023) as a practical route to improve fiber quality traits (Wang et al., 2023a). Large panel GWAS core QTL and revealed breeding footprints. Resequencing 318 worldwide upland accessions, Fang et al. mapped 119 loci: selection sweeps for lint yield centered on two ethylene pathway genes, whereas fiber length and strength alleles displayed a richer, less fixed spectrum (Fang et al. 2017). Such studies clarify why yield responded faster than quality in twentieth-century breeding. Diploid *G. arboreum* harbors fiber variants absent from tetraploids. Iqbal et al. (2021) performed GWAS on 215 accessions detected 7 429 significant SNP–trait links, distilled to 177 lead markers and 56 candidate genes many in cell wall biosynthesis or regulation and provides a ready reservoir for introgression. Similar surveys in *G. herbaceum* and *G. raimondii* are now underway, further broadening the cotton pangenome. Mapping with 115 interspecific introgression lines uncovered mostly small-effect (< 10% PVE) fiber QTL, showing that elite Upland cotton already carries many key alleles, while additional useful variants still reside in wild germplasm (Mangla et al. 2025).

Trait definition matters, since different ways of measuring the same trait can uncover different sets of loci. Using the AFIS single-fiber analyzer, Kim et al. (2024a, b) compared weight-based versus number-based length metrics. The weight metric yielded five QTL, but the number-based trait exposed a unique A13 locus (qFL-A13-1) that lengthened fibers without altering micronaire, a highly desirable combination for spinning. This case highlights how refined phenotyping can reveal loci invisible to traditional HVI measurements. Even with MAGIC and GWAS, most new loci explain only a sliver of variance. In the MAGIC study, 26 of 39 significant sites each accounted for < 5% PVE, and abundant epistatic interactions were required (Wang et al. 2023b) simultaneously estimating thousands of marker effects is now standard in elite breeding programs. Natural mutants are excellent sources for identifying genes involved in fiber development and their impact on fiber quality traits. Rong et al. (2005) mapped seven mutants related to seed fiber development, five of whi Naoumkina et al. 2022; ch were in the *G. hirsutum* background. Among these, *Li1* (Ligon lintless) and *Li2* affect fiber length, while *N1*, *Fbl* and *n2* result in fuzzless to lintless phenotypes (Rong et al. 2005). Building on this genetic mapping information, cotton researchers have fine-mapped the region, identified genes and causal mutations ranging from single-nucleotide changes to structural rearrangements, as well as cases involving small interfering RNA (siRNA)-mediated gene silencing (Naoumkina et al. 2022; Patel et al. 2020; Thyssen et al. 2017; Wan et al. 2016a, b). In addition, the genetic basis of other mutants targeting important fiber quality traits, like immature fiber (*im*) mutant and brown color cotton, has also been discovered (Hinchliffe et al. 2016; Thyssen et al. 2016). These studies demonstrate how natural mutants have advanced our understanding of genes and pathways that influence key fiber quality traits.

In addition to natural mutants, chemical mutagenesis, such as ethyl methane sulfonate (EMS), typically generates point mutations in the genome, providing novel germplasm that can be incorporated into breeding programs. These resources are valuable for improving agronomic traits and identifying the genes associated with them. EMS mutagenesis treatment in two upland cotton germplasms (Acala 1517–99 and TAM94L25) produced > 3 000 M₅ families; Patel et al. identified dozens with superior fiber traits (Patel et al. 2022, 2014). Backcrossing those mutations into an elite parent delivered progeny with ≈ 12% longer fibers or 22% stronger bundles, confirming that single-nucleotide changes can move polygenic traits (Patel et al. 2022). Physical mutagenesis offers yet another dimension: electron beam irradiation of TM-1 created mutant library displaying novel lint colors and ultrafine fibers, now feeding into QTL-seq pipelines (Zhao et al. 2022). With candidate genes in hand, CRISPR provides the fastest route from discovery to deployment. A 2024 synthesis lists more than two dozen fiber genes expansions, pectin lyases, MYB and ZF transcription factors already edited for proof-of-concept gains in length, strength or fineness (Baghyalakshmi et al. 2024; Fang et al. 2017). Tetraploid redundancy complicates knockout design, but multiplexed guides targeting homeolog pairs have begun to yield precise improvements with minimal off target effects. Three decades of QTL work have mapped a highly polygenic landscape punctuated by a few robust hotspots. Multi-omics now pinpoints causal genes; mutant libraries and CRISPR supply fresh alleles; and genomic selection aggregates their micro effects-. The remaining bottleneck is genetic diversity: many valuable loci reside in wild tetraploids or diploids, or still lie latent in mutant collections. By systematically introgressing, editing and stacking these alleles, breeders are poised to push cotton’s fiber quality beyond today’s ceiling fulfilling the promise first glimpsed when the very earliest QTL peaks appeared on linkage maps 30 years ago.

## Candidate genes and fiber developmental networks

Cotton fiber development is a tightly regulated, multistage process that begins with the differentiation of ovule epidermal cells into fiber initials and progresses through rapid cell elongation, secondary cell wall thickening and maturation. This complex process is controlled by an intricate network of transcription factors such as HDZIP IV proteins (Ding et al. 2020, 2015; Guan et al. 2008), MYB regulators (Machado et al. 2009; Pu et al. 2008; Walford et al. 2011) and TCP proteins (W. Li et al. 2017a, b; Ma et al. 2016) along with key metabolic enzymes involved in carbohydrate partitioning (Ruan et al. 2003; F. Tang et al. 2014a, b). Recent transcriptomic and genomic analyses have uncovered conserved hormonal signals including auxin and ethylene pathways that further refine the regulation of fiber cell fate and elongation, providing new targets for the genetic improvement of fiber quality.

### Phase 0: Pre‐initiation (− 5– − 1 DPA)

Five days before anthesis, an auxin minimum-to-maximum gradient first appears across the outer integument. This gradient is laid down by the PIN transporter *GhPIN3a*, and sharpened by the ROP GTPase *GhROP6*, which directs cells-specific turnover of PIN3a outside the future initiation spots (Hu et al. 2016; Xi et al. 2022). Low JA and BR titers keep the WD40–bHLH–MYB scaffold transcriptionally competent without prematurely activating it. The HDZIP IV genes GhHD1 and GhHD2 define positional identity. Their strong, L1-restricted expression at − 1 DPA defines cells that retain epidermal polarity: downregulating *GhHD1* retards both fiber and leaf trichome formation, whereas over-expressing it raises the density of fiber initials (Bai & Scheffler 2024; Guan et al. 2008; Wang et al. 2021).

Immediately downstream, MIXTA-like R2R3-MYBs, *GhMML3* (*GhMYB25-like_A12*) and *GhMML4* (*GhMYB25-like_D12*) commit ~ 15–20% of epidermal cells to a fiber fate. *GhMML3* is disrupted in the classic Naked-seed (N1) fuzzless mutant; RNAi silencing of *GhMML3* abolishes both lint and fuzz, whereas *GhMML4* knockdown selectively lowers lint density (Li et al. 2023; Walford et al. 2011; Wan et al. 2016a, b; Wu et al. 2018; Zhu et al. 2018). By − 1 DPA, the ovule is therefore “patterned” with a regular lattice of pre fiber cells but has not yet bulged outward.

### Phase I: fiber initiation (0–3 DPA)

Anthesis triggers a 12–18 h jasmonic acid surge that serves as a developmental gate. JA, in tandem with a burst of auxin and early BR, switches the WD40–bHLH–MYB complex from poised to active. *GhMYB25-like* rises first and, acting upstream of *GhMYB25*, launches trichome initiation genes; *GhMYB25-like* null mutants (*fl* or RNAi) produce completely fiberless seeds, while *GhMYB25* knockdowns merely shorten later elongation (Geng et al. 2023; Lee et al. 2006; Walford et al. 2011; Wan et al. 2016a, b; Wu et al. 2018, 2006; Zhao et al. 2024) (Fig. 2A). JA signaling must shut off quickly. *GhJAZ2* protein, induced by sustained JA, binds *GhMYB25-like*, *GhGL1* (a MYB), *GhMYC2* (a bHLH) and the WD40 partner, thereby choking the initiation complex; over-expressing *GhJAZ2* narrows the initiation interval and reduces both lint and fuzz (Guan et al. 2014; Hu et al. 2016).

Ethylene signaling is engaged through hormone cross talk at this stage. The R2R3-MYB *GhWER* peaks at − 1 to 0 DPA, directly activating *GhACS1* and *GhETR2* to create an ethylene minipulse essential for full initiation yield; CRISPR knockouts of *GhWER* markedly cut fiber number (Zhao et al. 2024). *GhACS6.3*, an ovule specific ACC synthase, supplements this pulse; its overexpression elevates ACC, boosts sucrose related transcripts and nearly doubles lint initials (Geng et al. 2023). By ~ 3 DPA, the number of lint initials per seed plateaus near 20,000; genotype specific fuzz initials follow. Subsequent fiber yield is therefore set chiefly by the allelic strength of *GhMML3/GhMML4* and the duration of the JA to ethylene relay (Bai & Scheffler 2024).

### Phase II: rapid elongation (3–20 DPA)

Primary wall expansion proceeds at 1–2 mm day⁻^1^, driven by a four-way hormone engine: GA, BR, auxin and ethylene. GA removes the DELLA repressor *GhSLR1* from *GhHOX3*, allowing the *GhHOX3–GhHD1* heterodimer to bind L1-box elements in expansin–like *GhRDL1* and *GhEXPA1*, plus aquaporins, thereby loosening the wall (Fu et al. 2023; Kabir et al. 2023; Shan et al. 2014; Wu et al. 2023). Silencing *GhHOX3* shortens fibers by ~ 80%, illustrating its centrality (Wang et al. 2023a, b; Xing et al. 2023).

BR output is tuned by a PRE–FP–ACE triHLH rheostat. Full bHLH-*GhACE1* activates *GhEXP8* and *GhPIP2;7*; atypical HLHs *GhFP1/2* bind and block *GhACE1*; small HLHs *GhPRE1/5*, induced by BR, sequester *GhFPs* and restore ACE1 activity (Li et al. 2023). *GhBES1* links BR back to GA: BR treatment or *GhBES1* overexpression raises active GA, whereas brassinazole lowers GA but can be partly rescued by exogenous GA (Hou et al. 2025). Strigolactones (SLs), a class of plant hormones best known for regulating shoot branching and root growth, also act as adjustable brakes on cotton fiber elongation. *GhSMXL7/8* bind *GhSLR1* (stabilizing DELLA), tether *GhHOX3* (blocking its DNA binding) and repress auxin responsive *GhARF18/19*. Rising SL triggers SCF^D3/MAX2-mediated degradation of SMXL7/8, releasing all three growth axes (Y. Sun et al. 2024). Consequently, silencing ARF18/19 shortens fibers, whereas SMXL knockdown lengthens them.

Auxin signaling stays high through mi elongation: ARF18/19, ARF2 and *GhGRF4* activate *GhGASA24* and other wall loosening targets. When auxin finally declines, Aux/IAA *GhIAA14* accumulates and binds *GhARF7*, preventing premature secondary wall genes (Y. Sun et al. 2024). Ethylene maintains turgor by upregulating sucrose transport and expansions yet is self-limited: *GhXB38D* (an E3 ligase) targets ACS4/ACO1, capping the ethylene peak as elongation wanes. Meanwhile, ABA climbs steadily after ~ 15 DPA. ABA activated kinases *GhCPK84/93* phosphorylate *GhSUS2*, diverting sucrose away from wall loosening and lowering cell turgor (Wang et al. 2023a, b). If ABA biosynthesis or perception is impaired, elongation persists beyond its usual 20 DPA ceiling; conversely, early ABA spikes truncate growth (Bai & Scheffler 2024).

Under optimal GA–BR-dominant balance, upland cotton fibers extend 3–4 cm (~ 30,000% their original length). Classic *Li1/Li2* mutants, defective in GA–*GhHOX3* signaling, terminate growth early and illustrate how a single lesion here can halve final length. Once ABA overtops auxin/GA, the transcriptome shifts toward cellulose synthase and secondary wall regulators, marking Phase III onset.

### Phase III: transition to secondary wall (≈ 20–25 DPA)

Hormone ratios invert: ABA peaks, auxin falls, GA/BR persist only moderately, and ethylene remains steady. A microRNA switch ensures the transition is irreversible. During elongation, *miR319* represses CIN-TCPs; when *miR319* declines, *GhTCP4* accumulates, represses further expansion and activates secondary wall TFs (Cao et al. 2020). Over-expressing a *miR319*-resistant *GhTCP4* provokes premature wall thickening and short fibers, confirming its gatekeeper role.

**Fig. 3:**
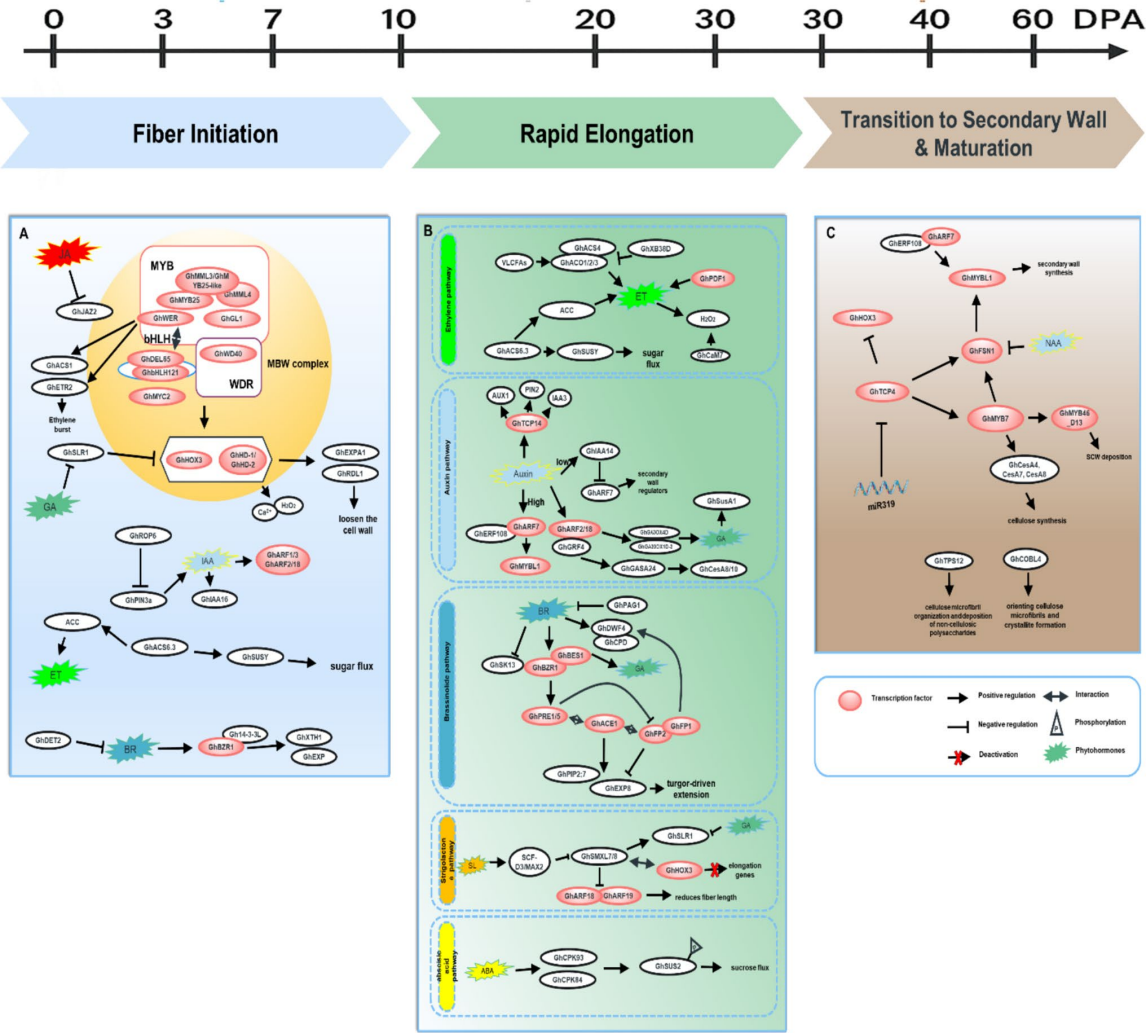
Schematic representation of the regulatory pathways linking key regulatory factors and phytohormones across three stages of cotton fiber development

*GhIAA14–GhARF7* provides an auxin-dependent checkpoint. High auxin keeps ARF7 free, but once auxin drops *GhIAA14* binds ARF7 and delays *CesA4/7/8* until elongation truly ceases (Yaru Sun et al. 2024). Ethylene then cooperates: GhERF108 partners with ARF7-1/-2 to coactivate GhMYBL1, which in turn drives SCW CesAs (Wang et al. 2023a, b). Simultaneously, cortical microtubules reorient from transverse to steep helices, guiding orderly microfibril layering. By ~ 25 DPA, length gain is negligible; cellulose now deposits at > 2 mg day⁻^1^ per fiber (Fig. 3).

(A) Fiber initiation stage, highlighting the MYB–bHLH–WDR transcriptional complex and associated proteins (yellow background).(B) Fiber elongation stage, illustrating the complex cross talk among multiple phytohormones, including abscisic acid (ABA), strigolactones (SL), brassinosteroids (BR), ethylene (ET), gibberellic acid (GA) and auxin, as well as sugar signaling and other small signaling molecules.(C) Fiber maturation stage, during which the levels of most phytohormones gradually decline. This stage is characterized by the transcriptional regulation of secondary cell wall (SCW)-specific cellulose synthase genes and other SCW-related genes by a cascade of transcription factors. Black arrows: Pointed arrows represent activation, whereas blunt-ended arrows indicate suppression.

### Phase IV: secondary wall thickening and maturation (25 DPA to open boll)

Abscisic acid (ABA) now dominates the hormonal landscape, sustaining progressive dehydration and inducing late embryogenesis abundant (LEA) proteins that protect the desiccating cytoplasm. A low, steady trickle of ethylene keeps sucrose import and basal metabolism active without retriggering growth. Cellulose deposition is driven by the secondary wall cellulose synthase complex encoded by *GhCESA4*, *GhCESA7* and *GhCESA8*. Their promoters are activated by the fiber-specific NAC master regulators *SECONDARY WALL NAC DOMAIN1/2* and by the R2R3-MYBs *GhMYBL1* and *GhMYB7*; over-expressing *GhMYB7* thickens the wall and boosts tensile strength but raises micronaire, whereas RNA-interference delays thickening and yields finer lint (Huang et al. 2021) (Fig. 2C).

Microfibril orientation and crystallinity are refined by *GhCOBL4*, a COBRA-like GPI-anchored protein whose elevated expression correlates with stronger, more crystalline fibers. Carbon allocation into the wall is further tuned by trehalose-6-phosphate signaling through *GhTPS12*, which subtly adjusts hemicellulose composition and, consequently, final fiber flexibility.

Wall hardening is finished by a Ca^2^⁺/ROS “varnish.” Ca^2^⁺-binding proteins most notably the calmodulin *GhCaM7* and several annexins regulate Capectate cross-link density, while class III peroxidases and alternative oxidases (AOXs) mediate phenolic cross-bridging. Early over-expression of *GhCaM7* elevates ROS and accelerates elongation (W. Tang et al. 2014a, b) conversely, misregulating peroxidase or AOX activity perturbs oxidative curing and shifts fineness or maturity (Xu et al. 2019). A second layer of control over fiber “finish” is supplied by late–acting transcription factors such as *GhFSN1* (Fiber Secondary wall NAC 1). *GhFSN1* directly upregulates *GhCESA4/7/8* and a suite of lignocellulose-related genes; prolonged GhFSN1 activity extends cellulose deposition and raises micronaire, whereas early downregulation leaves a thinner but often softer wall.

By dehiscence, each fiber is a dead, three to six micron walled cellulose tube whose length (fixed in Phase II) and wall traits (set in Phases III–IV) dictate bundle tenacity, micronaire and maturity. Breeders exploit this framework: boosting the CESA–NAC/MYB circuit or *GhCOBL4* enhances strength but risks coarser micronaire, whereas gently shortening Phase IV or dampening *GhMYB7/GhFSN1* can deliver finer, more uniform lint at some cost to tensile performance. Precision edits in upstream regulators *GhBES1* (BR → GA relay), *GhSMXL7/8* (SL brake) or *GhTCP4* (miR319‐timed switch) offer a rational route to tailor fiber quality across modern market niches, all while respecting the chronological gene network logic summarized here (Huang et al. 2021) (Supplementary Table 2).

## Homoeolog expression balance in polyploid cotton fiber

Allotetraploid cotton (*Gossypium hirsutum* and *Gossypium barbadense*) contains two homologous subgenomes derived from an A-genome ancestor (e.g., *Gossypium arboreum*) and a D-genome ancestor (e.g., *Gossypium raimondii*), such that most genes are present as two homoeologs (At and Dt). In modern cotton fiber, most genes are expressed from at least one subgenome; however, coexpression network analyses indicate that only about 20% of homoeologous gene pairs are found together within the same module, suggesting widespread subfunctionalization and divergent regulation (Grover et al. 2012; Yoo et al. 2013). Moreover, domesticated cotton fibers often exhibit a subtle bias in which many modules show higher expression of D-genome homoeologs even though the ancestral A-genome contributed spinnable fiber, and the D-genome progenitor produced only short fuzz (M. Wang et al.2017a, b). For example, in the β-tubulin gene family, while A-homoeologs may dominate in wild cotton fibers, domesticated cotton appears to utilize both A and D copies more equally or even preferentially the D copy to optimize microtubule dynamics during elongation (Wendel & Grover 2015). This balanced recruitment from both subgenomes is thought to provide the polyploid with a more robust genetic toolkit that has been refined by domestication, enabling superior fiber traits (Jareczek et al. 2023). Overall, these findings illustrate how polyploidy and subsequent regulatory divergence have allowed the fusion and re-partitioning of gene functions, with selection favoring an optimal balance of homoeolog expression in modern cotton fiber.

## Conclusions and future directions

Over the last decade, our understanding of the genetic basis of cotton fiber quality has advanced from mapping quantitative trait loci (QTL) on linkage maps to pinpointing specific genes and regulatory networks that underlie fiber development (Fang et al. 2017; Yoo & Wendel 2014). Domestication of cotton involved both predictable changes such as the selection of alleles for longer fibers in all four domesticated species and surprising convergences, for example, similar coexpression network “densification” observed in two independent polyploid domestications (Gallagher et al. 2020; Renny-Byfield et al. 2016). The parallel domestications in different *Gossypium* lineages provide natural replicates for comparison. Ongoing comparative genomics between G. hirsutum and *G. barbadense*, as well as between the Old-World diploids (*G. arboreum* versus *G. herbaceum*), will further illuminate whether the same pathways were targeted during selection or if distinct genetic routes led to similar fiber phenotypes (Applequist et al. 2001; Brubaker & Wendel 1994; Desai et al. 2008). Early evidence suggests that while both upland and Pima cotton domestications appear to have upregulated a common set of fiber elongation genes such as those encoding expansins and lipid transfer proteins, indicating parallel evolution differences in fuzz presence or seed surface traits imply that certain genes, perhaps members of the *GhMML* family involved in fuzz development, were altered in one domestication event but not the other (Walford et al. 2011; Zhang et al. 2015). Likewise, the Old-World diploids may have achieved spinnable fiber through distinct initial mutations, as suggested by differences in their repeat content and in some gene families (Abdul Kadir 1976; Gulati & Turner 1928).

Technologically, cotton research is now poised to leverage genome editing and multi-omics integration to validate candidate genes and even to create improved fiber genotypes by design. For example, CRISPR/Cas9 has been employed to knock out negative regulators such as by editing genes that suppress fiber elongation and to target both homoeologs simultaneously in allotetraploid cotton (Gao et al. 2017; C. Li et al. 2017a, b). In addition, pangenome studies and GWAS-seq are now underway to capture nearly all genetic variants across thousands of cotton accessions, including those from wild relatives, which will improve mapping resolution and likely reveal previously unknown alleles with large effects on fiber traits (Baghyalakshmi et al. 2024; Fang et al. 2017). Furthermore, epigenomic profiling of fiber cells examining DNA methylation and histone modifications has started to uncover epigenetic regulators that may explain some of the “missing heritability” of fiber quality (Tuttle et al. 2015).

From a breeding perspective, marker-assisted selection and genomic selection have increasingly been incorporated into cotton breeding programs. For instance, DNA markers linked to a major fiber length allele on chromosome D11 and a fiber strength allele on chromosome D02 are now routinely used to pyramid favorable alleles (Wang et al. 2023b; Zhang et al. 2016). In parallel, the integration of wild cotton germplasm into breeding through strategies such as bridge crosses, embryo rescue and even gene editing to remove incompatibility loci remains a promising route to reintroduce genetic diversity into modern cultivars. These approaches illustrate how the tremendous allelic variation present in wild relatives can be harnessed to further enhance fiber quality, resilience and sustainability in cotton production.

## Supplementary Information

Below is the link to the electronic supplementary material.

Supplementary file1 (PDF 414 kb)

Supplementary file2 (PDF 158 kb)

## Data Availability

All datasets discussed are publicly available in the cited literature or databases (e.g., CottonGen).
