# Supplementary material for: Genetic basis and role of exotic accessions in cultivated cotton fiber quality improvement
Source: Theor Appl Genet. 2025 Sep 27;138(10):260. doi: 10.1007/s00122-025-05043-2 (PMC12476451; doi:10.1007/s00122-025-05043-2)
Supplement: Supplementary file 1 — Supplementary file1 (PDF 414 kb) [file 122_2025_5043_MOESM1_ESM.pdf]

Supplementary Table 2. Experimentally validated genes affecting cotton fibre quality.

Gene	Species	Functional Role	Phase	Hormone Pathway(s)	Validation	Citation
14-3-3	*G. hirsutum*	Scaffold protein; modulates BES1/BZR1 nuclear shuttling	Initiation & Elong.	BR	Y2H & OE (exp.)	Zhou et al. 2015
BES1 (Gh_D02G0939)	*G. hirsutum*	BES1 TF linking BR→GA; boosts elongation	Elongation	BR → GA	OE	Hou et al. 2025
CERP	*G. hirsutum*	Cell-wall enzyme (pectate lyase); BR-BES1 target for elongation	Elongation	BR targets	ChIP-qPCR (exp.)	Zhu et al. 2023
CesA4 / CesA7 / CesA8	*G. barbadense*	Cellulose synthase genes: key for secondary cell-wall biosynthesis; in G. barbadense, these show delayed but stronger up-regulation, underlying extra-	SCW	–	Expression profiling (FPKM across DPA); comparative analysis vs. G. hirsutum	Liu, X., Zhao, B., Zheng, HJ. et al. 2015
GaFormins	*G. arboreum*	Formin family (FH1/FH2): regulate actin polymerization during elongation	Elongation	–	Transcriptome analysis showing dynamic AS and expression (correlative)	Shing, Pollob et al. 2024
GaGTs	*G. arboreum*	Trihelix TFs (e.g., GaGT15): induced during fiber initiation, likely regulate epidermal cell fate/outgrowth	Initiation	–	Genome-wide identification & qRT-PCR in initiation stage (correlative)	Prasad, Priti et al. 2022
GaHD-ZIP IV homologs	*G. arboreum*	HD-ZIP IV family members (e.g., GaHD1-like): regulate epidermal cell fate and initiation; diploid-specific expression patterns noted	Initiation	–	Transcript profiling; homology inference from G. hirsutum functions (correlative)	Hande, Atul S et al. 2017
GaMYB25-like	*G. arboreum*	MYB regulator homolog: likely controls fiber initiation; differential expression vs. non-fiber tissues	Initiation	–	Comparative transcript profiling in diploid fibers (correlative)	Walford, Sally-Ann et al. 2011
GaMYB25-like homolog	*G. arboreum*	MYB regulator homologous to G. hirsutum GhMYB25-like; likely controls fiber initiation but with possibly divergent regulation in diploid; identified in G.	Initiation	–	Comparative expression profiling; candidate from transcriptomic datasets	Walford, Sally-Ann et al. 2011
GaPEL48_Dt	*G. arboreum*	Pectate lyase-like: promotes fiber initiation via cell-wall modification	Initiation	–	Expression profiling; functional hints in cotton assays (correlative)	(inferred)

GaRBOH	*G. arboreum*	NADPH oxidase: ROS generation in fiber cells; roles in elongation signaling; expression patterns differ from tetraploid cotton	Elongation / Initiation	–	Expression profiling under fiber development stages (correlative)	Wang, Wei et al. 2020
GbEXP homologs	*G. barbadense*	Expansin family: cell-wall loosening during elongation; G. barbadense paralogs show distinct expression peaks correlating with prolonged elongation	Elongation	–	Expression profiling in long-fiber vs. short-fiber lines (correlative)	Wang et al., 2022
GbEXPA2	*G. barbadense*	α-Expansin paralog: promotes cell-wall loosening during fiber elongation; more highly expressed in extra-long fibers	Elongation	–	Expression profiling; transgenic/heterologous assays in Arabidopsis (exp.)	Li, Yang et al. 2016
GbEXPATR	*G. barbadense*	Species-specific expansin: enhances fiber elongation when overexpressed	Elongation	–	Expression profiling; functional assays (exp.)	Li, Yang et al. 2016
GbMYB46-like	*G. barbadense*	SCW master regulator homolog: directs CesA expression; delayed upregulation aligns with extended SCW deposition	SCW	–	Expression profiling; co-expression networks (correlative)	Wang et al., 2019
GbNTL9	*G. barbadense*	NAC transcription factor: positively regulates fiber strength in Pima cotton; allelic variants associate with stronger fibers	SCW / Maturation	–	Expression profiling; candidate functional validation in lines (correlative/exp.)	Wu, Mi et al. 2025
GbPEL76	*G. barbadense*	Pectate lyase-like: promotes cell-wall loosening during elongation, similar to homologs in G. hirsutum but with species-specific expression timing	Elongation	–	Expression profiling in fibers; some functional assays in model systems (correlative/exp.)	Meng, Qingying et al. 2025
GbPRE1	*G. barbadense*	PRE1 homolog: modulates cell elongation signaling; prolonged expression corresponds with extended elongation phase	Elongation	–	Comparative expression profiling vs. G. hirsutum (correlative)	Liu, X., Zhao, B., Zheng, HJ. et al. 2015
GbRBB1_A07	*G. barbadense*	RBB1 homolog: identified via GWAS/MAGIC in G. barbadense for superior fiber length/strength/uniformity	Likely Elongation / SCW	–	GWAS association in diverse populations; preliminary expression correlation	Zhang et al., 2011

GbSWEETs	*G. barbadense*	Sugar transporter family: several GbSWEETs highly expressed in elongating fibers, facilitating sucrose influx for turgor	Elongation	–	Genome-wide identification; expression patterns during elongation (correlative)	ZHAO, L., YAO, J., CHEN, W. et al. 2018
Gh_D06G1908	*G. hirsutum*	Candidate gene from GWAS for fiber quality traits (identified in QTL region)	Likely Elongation/SCW	–	GWAS + RNA-seq & RT-qPCR (correlative)	Liu, Wei et al. 2020
Gh_D09G2376	*G. hirsutum*	Candidate gene from GWAS for fiber quality traits (identified in QTL region)	Likely Elongation/SCW	–	GWAS + RNA-seq & RT-qPCR (correlative)	Liu, Wei et al. 2020
Gh14-3-3L	*G. hirsutum*	14-3-3 regulatory protein; involved in early elongation signaling	Elongation	–	Fiber-abundant expression at 6–9 DPA	Zhou, Ying et al. 2015
Gh3GT	*G. hirsutum*	Anthocyanidin 3-O-glucosyltransferase: modulates anthocyanin vs. PA flux; overexpression can shift fiber color	Fiber color; SCW	–	OE in brown cotton (exp.)	Liu et al. 2018; Canavar & Rausher 2021
GhACS6.3	*G. hirsutum*	ACC synthase; raises ethylene & sugar flux	Initiation	Ethylene	OE	Geng et al. 2023
GhAct1	*G. hirsutum*	Actin cytoskeleton protein; required for fiber elongation	Elongation	–	RNAi knockdown disrupts actin network, blocking elongation	Li, Xue-Bao et al. 2005
GhADF1	*G. hirsutum*	Actin-depolymerizing factor: regulates actin dynamics; down-regulation affects fiber properties (length, strength)	Elongation	–	RNAi/overexpression studies (exp.)	Wang, Hai-Yun et al.2009
GhAKT1	*G. hirsutum*	K^+ transporter; sustains turgor	Elongation	—	VIGS	Xu et al. 2014

GhANR	*G. hirsutum*	Anthocyanidin reductase: converts anthocyanidin → epicatechin (PA precursor); VIGS reduces pigment	Fiber color; SCW	–	VIGS in brown cotton (exp.)	Gao et al. 2019
GhAOC1	*G. hirsutum*	AOC paralog with fiber-stage expression.	Initiation	JA biosynthesis	Expression profiling; no direct functional test. (correlative)	Wang et al. (2015)
GhAOS2	*G. hirsutum*	AOS paralog upregulated around fiber initiation.	Initiation	JA biosynthesis	Expression profiling; no direct knockdown. (correlative)	Wang et al. (2015)
GhAOX	*G. hirsutum*	Alternative oxidase; modulates ROS levels in fibers, affecting wall curing and maturity.	Transition → SCW	ROS metabolism	Expression profiling + AOX manipulation in model assays (correlative)	Xu et al. 2019
GhAPX (e.g., ascorbate peroxidase)	*G. hirsutum*	ROS-scavenging enzyme: maintains H_2O_2 homeostasis during fiber development; influences cell-wall loosening and elongation	Elongation / Transition	–	Expression profiling; functional assays (exp./correlative)	Zhou, Ting et al. 2016
GhAPY (apyrase)	*G. hirsutum*	Nucleoside triphosphate-diphosphohydrolase: regulates extracellular nucleotides, influences fiber elongation	Elongation	–	Ovule culture assays; expression studies (exp./correlative)	Clark, Greg et al. 2010
GhARF18 / GhARF19	*G. hirsutum*	ARFs repressed by GhSMXL7/8; promote elongation genes when derepressed	Elongation	Auxin-responsive TF	CRISPR lines show reduced fiber length; ChIP-qPCR	Sun et al. 2024
GhARF2	*G. hirsutum*	Auxin Response Factor that (with GhGRF4) activates GhGASA24 → CesA8/10 for wall thickening	Late Elong. → Transition	Auxin-responsive TF	Y1H/ChIP; OE alters cell-wall thickness	Tian et al. 2024
GhARF7-1	*G. hirsutum*	Auxin-response factor partnering ERF108	Transition → SCW	Auxin	OE / Y2H	Wang et al. 2023
GhARF7-1 / GhARF7-2	*G. hirsutum*	Pair of ARFs that interact with GhERF108 to induce GhMYBL1 & CesA genes	Transition → SCW	Auxin-responsive TF	OE, Y2H, ChIP; double-RNAi delays SCW onset	Wang et al. 2023
GhARF7-2	*G. hirsutum*	Redundant with GhARF7-1	Transition → SCW	Auxin	OE / Y2H	Wang et al. 2023

GhAUX1-like	*G. hirsutum*	Auxin-influx carrier facilitating IAA uptake during rapid elongation	Elongation	Auxin transport (influx)	Over-expression increases fiber length; radiolabelled IAA uptake	Sun et al. 2021
GhBEE family	*G. hirsutum*	BEE-like bHLH TFs responsive to BR/GA; beyond GhBEE3, other homologs may regulate elongation or stress responses in fibers.	Elongation	BR/GA crosstalk	Expression profiling; some OE/RNAi in ovule culture (correlative/exp.)	Chen et al., 2017
GhBEE3	*G. hirsutum*	bHLH TF ("Brassinosteroid Enhanced Expression 3" homolog); implicated in early fiber cell regulatory network from spatial data.	Early Initiation	BR-related TF	Spatial transcriptome + network inference (correlative)	Sun, X., Qin, A., Wang, X. et al. 2025
GhBES1	*G. hirsutum*	Canonical BES1 TF; activates BR-responsive elongation genes (e.g. CERP, SMO2-2)	Elongation	BR	OE / CRISPR (exp.)	Zhu et al. 2023
GhBES1.4	*G. hirsutum*	Fiber-enriched BES1 isoform; up-regulates GhKCS10, GhEXL3, GhKRP6, GhCYP84A1, GhHMG1	Elongation	BR→GA crosstalk	OE & CRISPR (exp.)	Yang et al. 2023
GhBG	*G. hirsutum*	β-1,4-Glucanase (endo-glucanase); loosens primary wall & facilitates SCW assembly	Secondary CW	–	Expressed during elongation; hydrolyzes cellulose/pectin	Ma, Guo-Jia et al. 2006
GhbHLH282	*G. arboreum*	bHLH TF; positively regulates fiber elongation downstream of BR	Elongation	BR targets	OE & VIGS (exp.)	Lu et al. 2018
GhBIN2	*G. hirsutum*	Negative BR kinase; phosphorylates BES1/BZR1	— (regulatory node)	BR	Protein interaction & enzyme assay (exp.)	Zhu et al. 2023
GhBLH1	*G. hirsutum*	GA-released BLH TF; activates KCS12/VLCFA route	Elongation	GA	OE	He et al. 2024
GhBOP1	*G. hirsutum*	Mediates SUMOylation of GhBES1; influences fiber length and plant height via BR signaling modulation.	Elongation	BR signaling / post-translational	OE/RNAi + SUMO assays + phenotyping (exp.)	Wang, Bingting et al. 2024
GhBRI1	*G. hirsutum*	Cell-surface BR receptor; perception of BR signal in ovule epidermis	Initiation → Elongation	BR core signalling	VIGS & OE (exp.)	Sun et al. 2015
GhBZR1	*G. hirsutum*	BR-responsive BES1/BZR1 TF; promotes initiation	Initiation	Brassinosteroid	Expression in ovules; implied role in initiation	Zhou, Ying et al. 2015

GhBZR3	*G. hirsutum*	BZR1-like TF; activates GhKCS13 (VLCFA synthesis)	Elongation	BR	OE & EMSA (exp.)	Shi et al. 2022
GhCalS / GhCallose synthase	*G. hirsutum*	Callose deposition/regulation at plasmodesmata: influences symplastic isolation during elongation	Elongation initiation	–	Expression profiling; inference from Arabidopsis (correlative)	Feng, Jiajia et al. 2021
GhCAM7	*G. hirsutum*	Calmodulin-like protein affecting ROS and Ca^{2+} signaling; overexpression can increase ROS and influence elongation duration.	Elongation	Ca^{2+}/ROS signaling	OE in fiber cells + ROS assays (exp.)	Tang, Wenxin et al. 2014
GhCBP60 / GhSARD1	*G. hirsutum*	Homologs of SA biosynthesis regulators (Calmodulin-binding proteins controlling ICS expression). Expression in cotton tissues noted; potential modulators of SA levels in fibers under certain stimuli.	Initiation → Elongation	SA biosynthesis regulation	Homology inference; expression data from transcriptomes; no direct fiber functional tests (predicted)	Luo, Kun et al. 2024
GhCCD7 / GhCCD8	*G. hirsutum*	Carotenoid cleavage dioxygenases in SL biosynthesis; catalyze steps to produce SL precursors	Elongation / Transition	SL biosynthesis	Expression profiling in fibers; functional inference from Arabidopsis homologs; some VIGS/OE in ovule assays suggest roles in SL level and fiber traits (correlative/exp.)	Ma, Jianhui et al. 2025
GhCesA1 (CesA)	*G. hirsutum*	Cellulose synthase (homologous to bacterial celA); synthesizes cellulose in SCW	Secondary CW	–	cDNA expression high during SCW stage; in vitro fragment binds UDP-glucose	Jacob-Wilk, Debora et al. 2006
GhCesA3	*G. hirsutum*	Cellulose synthase catalytic subunit; active in SCW biosynthesis	Secondary CW	–	Fiber-specific expression in SCW stage	

GhCesA4	*G. hirsutum*	Cellulose synthase catalytic subunit; a core component of the cellulose synthase complex responsible for synthesizing cellulose during secondary cell-wall thickening in fibers.	SCW (~16–25 DPA)	Regulated by SCW TFs (e.g., GhMYB7, GhMYBL1); responsive to ABA signaling; may be influenced indirectly by GA/BR/ethylene via upstream TFs.	Expression and protein profiling in fibers; promoter binding by GhMYB7/GhMYBL1 shown; RNAi/CRISPR delays SCW and alters wall thickness (exp.).	Huang, Junfeng et al. 2021
GhCESA6 / GhCSLC (putative)	*G. hirsutum*	Cellulose synthase-like genes: may contribute to primary wall or glucomannan synthesis affecting elongation	Elongation / SCW	–	Expression profiling; some functional inference (correlative)	
GhCesA7	*G. hirsutum*	Cellulose synthase catalytic subunit; partners with GhCesA4 and GhCesA8 in the CSC to polymerize cellulose microfibrils during fiber secondary wall deposition.	SCW (~16–25 DPA)	Regulated by SCW TFs (GhMYB7, GhMYBL1) and responsive to ABA (upregulation under ABA treatment); part of SCW network influenced by NAC/MYB cascades.	Transcript and protein expression profiling; promoter binding by GhMYB7/GhMYBL1; ABA-induced upregulation observed; functional assays delay SCW when silenced (exp./correlative).	Huang, Junfeng et al. 2021

GhCesA8	*G. hirsutum*	Cellulose synthase catalytic subunit; works with GhCesA4 and GhCesA7 in the cellulose synthase complex to produce the bulk cellulose in the secondary cell wall of fibers.	SCW (~16–25 DPA)	Regulated by SCW TFs (GhMYB7, GhMYBL1); responsive to ABA signaling; integrated into SCW transcriptional network (NAC/MYB modules).	Expression and protein profiling in fibers; promoter binding by GhMYB7/GhMYBL1 demonstrated; ABA-induced expression changes; RNAi/CRISPR affects SCW deposition (exp./correlative).	Huang, Junfeng et al. 2021
GhCHI (GhCHI-1, GhCHI-2)	*G. hirsutum*	Chalcone isomerase: catalyzes chalcone → naringenin; modulates pigment flux; silencing alters fiber color and affects fiber length/micronaire	Fiber color; initiation/elongation	–	RNAi in brown cotton (exp.)	Liu et al. 2018; Tan et al. 2013
GhCHS (e.g., GhCHS2)	*G. hirsutum*	Chalcone synthase: entry enzyme of flavonoid pathway; essential for proanthocyanidin/anthocyanin biosynthesis in colored fibers	Fiber color (SCW stage; ~20–30 DPA)	–	VIGS/RNAi in colored cotton (exp.)	Gao et al. 2019; Liu et al. 2018
GhCKI1 (CK-inducible genes)	*G. hirsutum*	Downstream CK-responsive genes; may mark CK signaling activity in ovule; roles in cell division or metabolism in ovule but not directly fiber expansion	Pre-initiation	CK response	Expression profiling under CK treatment in ovule culture; no direct fiber phenotype data (correlative)	Buck, Sarah H et al. 2009
GhCKX (e.g., GhCKX2, GhCKX3)	*G. hirsutum*	Cytokinin oxidase/dehydrogenase; degrades active CK; modulates CK homeostasis in ovule/fiber contexts; potential negative regulator of CK level	Initiation	CK catabolism	Expression profiling; functional characterization in other species; cotton-specific RNAi/OE in ovules limited (correlative)	Hu, Wei et al. 2025

GhCPC	*G. hirsutum*	CAPRICE-like MYB; negative regulator of initiation	Initiation	–	Detected in ovule epidermis; represses initiation	Liu, Bingliang et al. 2015
GhCPD	*G. hirsutum*	Cytochrome P450 in BR biosynthesis	Elongation	BR biosynthesis	Expression + VIGS (exp.)	Liu et al. 2020
GhCPK84	*G. hirsutum*	Ca^{2+}-DPK that phosphorylates SUS2 under ABA	Late Elong.	ABA	Phospho-proteomics + OE	Wang et al. 2023
GhCPK84 / GhCPK93	*G. hirsutum*	Ca^{2+}-dependent protein kinases induced by ABA; phosphorylate GhSUS2 to alter sucrose metabolism, inhibiting elongation	Late Elongation	ABA signaling	Phosphoproteomics and OE: increased protein levels under ABA; mutation/overexpression shows impact on fiber length (exp.)	Wang et al. 2023
GhCPK93	*G. hirsutum*	Redundant with GhCPK84	Late Elong.	ABA	Phospho-proteomics + OE	Wang et al. 2023
GhCrRLK1L family (e.g., GhCrRLK1L-x)	*G. hirsutum*	Catharanthus roseus RLK1-like receptor kinases; several members preferentially expressed at different fiber stages, possibly modulating cell-wall integrity or signaling.	Initiation → Elongation	RLK signaling / cell-wall sensing	Genome-wide ID + expression profiling (correlative)	Dongyun Zuo et al. 2024
GhCYP701A1	*G. hirsutum*	P450 involved in GA biosynthesis (ent-kaurene oxidase step); silencing impairs GA levels and fiber development	Initiation → Elong.	GA biosynthesis	RNAi/ VIGS reduces GA content and fiber elongation; expression correlates with GA biosynthesis stages (exp.)	Liang et al., 2025
GhCYP707A	*G. hirsutum*	ABA 8'-hydroxylase in other contexts; likely involved in ABA catabolism in fibers; expression patterns suggest role in modulating ABA turnover	Elongation → Transition	ABA catabolism	Homology-based inference; expression profiling indicates upregulation when ABA levels need reduction; functional tests pending (correlative)	Kong, Xiangqiang et al. 2016

GhCYP84A1	*G. hirsutum*	Cytochrome P450; cell-wall phenolic pathway, BES1.4 target	Elongation	BR targets	OE & ChIP (exp.)	L. Liu et al. 2023
GhD27	*G. hirsutum*	Carotenoid isomerase in SL biosynthesis; contributes to production of SL precursors; induced by GA–GRF4 module	Elongation / Transition	SL biosynthesis	Expression profiling: GA treatment upregulates GhD27; RNAi in ovule culture reduces SL levels and impairs fiber elongation (exp./correlative)	Tian, Zailong et al.2022
GhD53	*G. hirsutum*	SL signaling repressor homolog (DWARF53-like); suppresses fiber elongation by repressing target genes (e.g., GhFAD3); degraded in presence of SL to permit elongation	Elongation	SL signaling	Expression and functional assays: overexpression inhibits elongation; SL treatment promotes degradation, relieving repression (exp.)	Wang et al., 2025
GhDET2	*G. hirsutum*	Steroid 5α-reductase in BR biosynthesis	Initiation & Elong.	BR biosynthesis	OE in Arabidopsis (heterologous exp.)	Luo et al. 2007
GhDFR	*G. hirsutum*	Dihydroflavonol 4-reductase: late enzyme in flavonoid pathway; critical for anthocyanidin/proanthocyanidin synthesis in colored cotton	Fiber color; SCW	–	VIGS and expression profiling (exp.)	Zheng et al. 2023
GhDOX2	*G. hirsutum*	Dioxygenase identified via spatial transcriptomics; potentially regulates lipid or hormone metabolites in early fiber cells.	Early Initiation	Metabolism / signaling intermediates	Spatial transcriptome + co-expression (correlative)	Sun, X., Qin, A., Wang, X. et al. 2025
GhDWF4	*G. hirsutum*	BR C-22 hydroxylase; rate-limiting for BR	Elongation	BR biosynthesis	Expression + VIGS (exp.)	Liu et al. 2020
GhDXS6D	*G. hirsutum*	1-deoxy-D-xylulose-5-phosphate synthase; upstream in MEP pathway supplying carotenoid/ABA precursors	Transition → SCW	ABA biosynthesis (indirect)	OE increases carotenoids and ABA in developing fibers (exp.)	Chuannan Wang et al.2025

GhE6	*G. hirsutum*	Early lipid-transfer protein marker	Initiation → Early Elong.	—	RNAi	Lee et al. 2007
GhEDS5	*G. hirsutum*	Enhanced disease susceptibility 5 homolog; involved in SA transport from chloroplast to cytosol in Arabidopsis. Cotton homologs expressed in various tissues; potential role in SA distribution in fibers under stress.	Elongation	SA transport	Homology-based inference; expression profiling indicates presence but no fiber-specific functional data (predicted)	Hu, Lanxi et al. 2024
GhERF (putative downstream TFs)	*G. hirsutum*	Transcription factors transmitting SL signals to cell wall or metabolic genes; specific members identified in transcriptomic analyses under SL treatment	Elongation / SCW	SL signaling	Transcriptome plus ChIP prediction; limited direct validation in fiber context (correlative)	Liu, Chunxiao, and Tianzhen Zhang. 2017
GhERF family members (other than GhERF108)	*G. hirsutum*	AP2/ERF TFs involved in ethylene or stress signaling; some regulate SCW genes or cross-talk with auxin/ABA in fibers.	Elongation → SCW	Ethylene / SCW	Expression in fibers; a few characterized by ChIP or OE (correlative/exp.)	Liu, Chunxiao, and Tianzhen Zhang. 2017
GhERF108	*G. hirsutum*	ERF TF; with ARF7s activates MYBL1 & CesAs	Transition → SCW	Ethylene & Auxin	OE + ChIP	Wang et al. 2023
GhEXL3	*G. hirsutum*	Extensin-like; cell-wall loosening downstream of BES1.4	Elongation	BR targets	CRISPR KO & ChIP (exp.)	Zhang et al. 2024
GhEXP	*G. hirsutum*	Expansin family (GhEXP8/9); BR-responsive wall loosening	Rapid Elong.	BR targets	OE & promoter-binding (exp.)	Sun et al. 2005
GhEXPA1	*G. hirsutum*	α-expansin; loosens cell wall for elongation	Elongation	–	OE (with partner GhRDL1) dramatically increases fiber elongation and yield	Shan et al., 2014
GhEXPA3-1	*G. hirsutum*	α-Expansin; up-regulated by BES1, promotes cell expansion	Elongation	BR-regulated	Transcriptome + qRT-PCR (exp.)	Zhu et al. 2023
GhF3H	*G. hirsutum*	Flavanone 3-hydroxylase: mid-pathway in flavonoid biosynthesis; influences pigment accumulation; altered expression affects fiber properties	Fiber color; elongation	–	RNAi/expression studies (exp./correlative)	Tan et al. 2013; Canavar & Rausher 2021

GhFAD3	*G. hirsutum*	Linolenic acid biosynthesis enzyme repressed by GhD53; influences membrane lipid composition during elongation	Elongation	SL downstream target	Expression and promoter assays: GhD53 binds GhFAD3 promoters; overexpression/knockdown affects fiber elongation (exp.)	Wang, Huiqin et al.2025
GhFP1	*G. hirsutum*	Atypical HLH; early BR target that accelerates rapid elongation	Early Elongation	BR targets	OE & VIGS (exp.)	Liu et al. 2020
GhFP2	*G. hirsutum*	HLH that fine-tunes elongation (part of PRE/FP/ACE rheostat)	Elongation	BR targets	OE / CRISPR (exp.)	Lu et al. 2022
GhGA20ox1	*G. hirsutum*	GA 20-oxidase; catalyzes biosynthesis of bioactive GA in ovules/fibers; OE increases GA levels, fiber initials and elongation	Initiation → Elong.	GA biosynthesis	Overexpression in cotton: increased initials & elongation; measured GA levels (exp.)	Xiao et al. 2010
GhGA20ox2 / GhGA20ox3	*G. hirsutum*	Paralogs of GA 20-oxidase; likely contribute similarly to GA biosynthesis during fiber development	Initiation → Elong.	GA biosynthesis	Expression profiling in developing fibers/ovules; some OE studies in ovule culture (exp./correlative)	Wu et al. 2010
GhGA2ox	*G. hirsutum*	GA 2-oxidase; deactivates bioactive GA; modulates GA levels to fine-tune elongation	Elongation → Transition	GA catabolism	Expression profiling shows stage-specific patterns; functional studies pending (correlative)	Shi eta l., 2019
GhGA3ox	*G. hirsutum*	GA 3-oxidase; converts GA precursors to active GAs in fibers	Elongation	GA biosynthesis	Expression enriched during fiber elongation; inferred from transcriptomes; OE/CRISPR not yet reported in fiber context (correlative)	Zhu et al., 2021

GhGASA10-1	*G. hirsutum*	GAST1 homolog; promotes fiber elongation (cellulose synthesis)	Elongation	Auxin (IAA)	Upregulated by IAA; OE in Arabidopsis increases cell elongation	Chen, Baojun et al. 2021
GhGASA24	*G. hirsutum*	Auxin-responsive; modulates CesA8/10 & wall thickness	Elong. → Transition	Auxin	OE + ChIP	Tian et al. 2024
GhGH3.5	*G. barbadense*	IAA-amido synthetase; conjugates excess IAA, moderating elongation	Elongation	Auxin homeostasis (conjugation)	RNAi lengthens fibers; enzyme assays	Zhao et al. 2020
GhGID1-1	*G. hirsutum*	GA receptor showing ovule-predominant expression and feedback regulation by GA; may modulate initiation sensitivity	Initiation	GA perception	Expression profiling; GA treatment downregulates GhGID1-1 in ovules (exp./inferred)	Dong et al., 2009; Sun et al. 2008
GhGID1-1 … GhGID1-6	*G. hirsutum*	GA receptor homologs; bind GA to trigger DELLA degradation; some show ovule-predominant expression and GA-responsive regulation	Initiation → Elongation	GA perception	Cloned and expression profiled; GA-binding assays in vitro; expression inhibited by GA in ovules (exp./predicted role)	Sun et al. 2008
GhGL1 / GhMYB23	*G. hirsutum*	MYB proteins analogous to Arabidopsis GL1; involved in initiation complex; interact with JAZs in JA pathway.	Initiation	JA–MYB crosstalk	Y2H shows interactions with some GhJAZs; expression in ovule epidermis. (molecular interaction)	Li et al. (2008, 2011); Hu et al. (2016)
GhGRF4	*G. hirsutum*	Growth-regulating factor induced by GA; coordinates with strigolactone pathways and cell expansion factors to promote elongation	Elongation	GA signaling (crosstalk)	Expression upregulated by GA; OE increases fiber length; interaction with SL components shown; GA treatment induces GhGRF4 (exp.)	Sun et al. 2024; Tin et al., 2022;Tian et al., 2024

GhHB14_D10	*G. hirsutum*	HD-ZIP III TF; silencing enhances elongation but reduces wall thickness and cellulose content, indicating role in SCW regulation.	Elongation → SCW	HD-ZIP III / SCW	VIGS silencing + phenotyping (exp.)	Li, S., Yu, M., Qanmber, G. et al. 2024
GhHB7	*G. hirsutum*	HD-ZIP I/II family member; expression peaks during elongation; may influence cell expansion via stress or developmental signaling.	Elongation	HD-ZIP signaling	Expression profiling + promoter analysis (correlative)	Ahmad, Adeel et al. 2024
GhHD1	*G. hirsutum*	Homeodomain IV TF; required for fiber initiation	Initiation	–	RNAi knockdown delays initiation	Shan et al., 2014
GhHK (AHK homologs)	*G. hirsutum*	Cytokinin receptors (histidine kinases); perceive CK in ovule tissues; likely regulate ovule development but not directly fiber cell expansion	Pre-initiation → Initiation	CK perception	Homology-based identification; expression detected in ovules; no reverse genetics in fiber context reported (predicted)	Nishimura, Chika et al. 2004
GhHMG1	*G. hirsutum*	3-Hydroxy-3-methylglutaryl-CoA reductase; sterol precursor, BES1.4 target	Elongation	BR targets	OE & ChIP (exp.)	L. Liu et al. 2023
GhHOS3.7	*G. hirsutum*	Candidate gene from spatial metabolome/transcriptome; may relate to stress/metabolic adjustments in early fiber cells.	Early Initiation	Metabolism / stress response	Spatial transcriptome + metabolome (correlative)	Sun et al., 2025
GhHOX3	*G. hirsutum*	HD-ZIP IV TF; major regulator of fiber elongation	Elongation	GA (interacts with DELLA)	Silenced lines have very short fibers	Shan et al. 2014; Bai et al. 2024 pmc.ncbi.nlm.nih.gov
GhHOX4	*G. hirsutum*	HD-ZIP IV TF; promotes fiber elongation	Elongation	–	Silencing shortens fibers; OE lengthens fibers	Wang, Na-Na et al. 2024

GhHP (histidine phosphotransfer proteins: GhHP2, GhHP3, GhHP18, GhHP23, GhHP24, GhHP28, etc.)	*G. hirsutum*	Phosphotransfer intermediates in two-component CK signaling; several (e.g., GhHP3) show ovule/fiber-stage expression, implying roles in signaling during early fiber development	Initiation → Early Elongation	CK signaling	Genome-wide identification & expression profiling (RNA-seq, qRT-PCR across DPA stages) shows stage-specific expression; limited VIGS in stress context; predicted function in fiber from expression (moderately validated)	Zhao, L., Sun, L., Guo, L. et al. 2022
GhIAA14	*G. hirsutum*	Aux/IAA repressor; times SCW onset via ARF7	Transition → SCW	Auxin	CRISPR	Guo et al. 2025
GhICS1 / GhICS2	*G. hirsutum*	Isochorismate synthase(s); catalyze conversion of chorismate to isochorismate in SA biosynthesis. Expression detected in ovule/fiber tissues; may influence local SA levels under stress or developmental cues.	Initiation → Elong.	SA biosynthesis	Expression profiling in fiber stages (RNA-seq/qPCR); no direct knockouts reported (correlative)	Guo, Zhan, et al.2023
GhIPT (e.g., GhIPT1)	*G. hirsutum*	Isopentenyltransferase; catalyzes rate-limiting step in CK biosynthesis; OE elevates CK levels in ovules but has no significant effect on fiber initiation or quality	Initiation	CK biosynthesis	Overexpression in cotton (35S or seed-specific promoter): increased CK but no fiber phenotype (exp.)	Zhu et al. 2018
GhJAZ family (other members)	*G. hirsutum*	Multiple JAZ paralogs; some expressed in fibers. They likely fine-tune JA responses during initiation/early elongation.	Initiation → Elongation	JA signaling	Genome-wide identification: 30 GhJAZs; expression profiling shows stage-specific patterns; few individual functional tests besides GhJAZ2. (mostly correlative)	Li et al. (2017); Chen et al. (2019)

GhJAZ2	*G. hirsutum*	Jasmonate ZIM-domain repressor that binds and inhibits initiation TF complexes (GhMYB25-like, GhGL1, GhMYC2, GhWD40), blocking lint and fuzz initiation when overexpressed.	Initiation	JA signaling	Overexpression (OE) inhibits initiation and reduces fiber length; Yeast two-hybrid (Y2H) shows interactions with initiation TFs; expression peaks around −1 to +1 DPA. (well validated)	Hu et al. 2016; Haiyan et al. 2016
GhKCS (e.g., GhKCS13)	*G. hirsutum*	Very-long-chain fatty acid synthase genes activated downstream of SL signaling in some contexts; contribute to cell membrane/wall composition during elongation	Elongation	SL downstream effectors	Expression profiling shows induction by SL treatments; functional assays in fiber culture suggest roles in elongation and cell wall (correlative/exp.)	Shi et al. 2022
GhKCS10	*G. hirsutum*	VLCFA synthase; BES1.4 target for elongation	Elongation	BR targets	OE & ChIP (exp.)	Z. Yang et al. 2023
GhKCS13	*G. hirsutum*	3-Keto-acyl-CoA synthase for VLCFA synthesis; BZR3 target	Elongation	BR targets	OE & EMSA (exp.)	Shi et al. 2022
GhKCS19.4	*G. hirsutum*	Very-long-chain fatty acid synthase family member; identified in early fiber spatial/metabolome study—may affect membrane or cuticle.	Early Initiation	Lipid metabolism	Spatial transcriptome + metabolome correlation	Sun, X., Qin, A., Wang, X. et al. 2025
GhKRP6	*G. hirsutum*	Cyclin-dependent kinase inhibitor; promotes cell expansion downstream of BES1.4	Elongation	BR targets	CRISPR & ChIP (exp.)	Gu et al. 2023
GhLAR	*G. hirsutum*	Leucoanthocyanidin reductase: produces catechin units in proanthocyanidin pathway; VIGS reduces brown pigment	Fiber color; SCW	–	VIGS in brown cotton (exp.)	Gao et al. 2019
GhLAX/LAX-like	*G. hirsutum*	Auxin influx carriers beyond GhAUX1-like; some paralogs expressed in fiber tissues, likely modulating local auxin levels.	Elongation	Auxin transport	Expression profiling; homology inference (correlative)	Grover et al., 2025

GhLOG (LONELY GUY family)	*G. hirsutum*	Cytokinin-activating enzymes converting inactive CK nucleotides to active free bases; expression in ovules may affect local CK levels	Initiation	CK activation	Expression profiling suggests some GhLOG paralogs expressed in ovules; no fiber-specific reverse genetics yet (correlative)	Wang, Rong et al. 2021
GhLOG-like / GhIPT-like expression regulators	*G. hirsutum*	Transcriptional regulators or miRNAs modulating IPT/LOG expression in ovules; potential influence on CK biosynthesis during fiber initiation window	Initiation	CK regulation	Inferred from expression co-expression networks; candidate status; functional tests pending (predicted)	Wang, Rong et al. 2021
GhLOX2	*G. hirsutum*	Specific 13-LOX member with elevated expression at initiation.	Initiation	JA biosynthesis	RNA-seq correlation with fiber initiation; no direct mutant. (correlative)	Wang et al. (2015)
GhLTP3	*G. hirsutum*	Lipid transfer protein; contributes to cutin and cuticular formation during PCW (early elongation)	Elongation	–	Ovule/fiber-specific expression; implicated in wall formation	Deng, Jinwu et al. 2022
GhMADS11	*G. hirsutum*	MADS-box TF; positive in elongation	Elongation	–	Upregulated during elongation; functional studies pending	Li, Yang et al. 2011
GhMADS14	*G. hirsutum*	MADS-box TF; negative regulator in elongation	Elongation	–	Expression pattern suggests role; transgenics show shorter fibers when overexpressed	Zhou, Ying et al. 2014
GhMAP20L5	*G. hirsutum*	Microtubule-associated protein: regulates microtubule stability; suppression reduces fiber elongation rate, length, lint percentage	Elongation	–	VIGS suppression / phenotype (exp.)	Song et al., 2023
GhMAPKs / GhCDPKs (other than CPK84/93)	*G. hirsutum*	Kinases involved in stress/ABA/ROS signaling; may modulate fiber maturation under stress	Transition / Maturation	–	Expression profiling; some functional tests (correlative)	Wang, Meimei et al. 2007<br>Lv, Wen-Ben et al. 2024

GhMAX1	*G. hirsutum*	Cytochrome P450 involved in SL biosynthesis downstream of CCD7/CCD8; modulates SL levels influencing repression release	Elongation / Transition	SL biosynthesis	Expression detected in developing fibers; functional studies limited; inference from Arabidopsis/rice MAX1 roles (correlative)	Zailong Tian et al. 2022
GhMAX2	*G. hirsutum*	F-box protein receptor component; mediates ubiquitination of SMXL repressors (GhSMXL7/8) upon SL perception	Elongation	SL perception/signaling	VIGS and expression assays: silencing reduces SL-mediated effects on elongation; interacts with SMXL proteins (exp.)	Sun, Yaru et al. 2025
GhMAX3 / GhMAX4	*G. hirsutum*	Equivalent to CCD7/CCD8 in SL biosynthesis pathway nomenclature; modulate SL levels affecting SMXL degradation	Elongation / Transition	SL biosynthesis	Expression data; limited reverse-genetics in fiber context; inference from functional conservation (correlative)	Dong, Jie et al. 2024
GhMML3 (MYB)	*G. hirsutum*	MYB-MIXTA-like TF; promotes initiation of fuzz fibers	Initiation	–	Expressed in initiating ovules	
GhMML3 duplicates	*G. hirsutum*	Multiple paralogs (e.g., GhMML3_A and GhMML3_D) coordinate lint and fuzz initiation; redundancy and divergence important for fiber fate.	Initiation	MYB initiation module	Genetic analysis of duplicates + expression (exp.)	Rui Chen et al. 2025
GhMML4 (MYB)	*G. hirsutum*	MYB-MIXTA-like TF; promotes initiation of lint fibers	Initiation	–	Expressed in initiating ovules	Wang, Na-Na et al. 2024
GhMYB109	*G. hirsutum*	R2R3 MYB TF; promotes both initiation and elongation	Initiation, Elongation	–	Expression enriched in fibers	Pu L et al. 2008
GhMYB25	*G. hirsutum*	R2R3 MYB TF; positive regulator of fiber initiation	Initiation	–	Overexpression enhances initiation	Machado, Adriane et al. 2009

GhMYB25-like	*G. hirsutum*	R2R3-MYB "master switch" for initiation; targeted by GhJAZ2 repression.	Initiation	JA–MYB crosstalk	RNAi/mutant yields fiberless seeds; Y2H shows GhJAZ2 binds GhMYB25-like. (well validated)	Hu et al. (2016)
GhMYB25-like dominant negative	*G. hirsutum*	A dominant-negative mutation variant affecting fuzz vs. lint initiation, revealing compensatory initiation pathways.	Initiation	MYB initiation	Genetic mutant characterization (exp.)	Guannan Zhao et al. 2024
GhMYB4	*G. hirsutum*	Bifunctional TF: represses elongation and promotes SCW synthesis; coordinates the transition between elongation and SCW phases.	Transition	MYB / elongation–SCW switch	OE/CRISPR + transcriptome + ChIP (exp.)	Qiao, Lu et al.2025
GhMYB46-like	*G. hirsutum*	Homolog of Arabidopsis MYB46; potential master regulator of SCW biosynthesis in fibers.	SCW	MYB / SCW master regulator	Expression profiling + inferred from SCW studies (correlative)	Zhong, Ruiqin, and Zheng-Hua Ye. 2012
GhMYB46-like / GhMYB83-like / GhVND homologs	*G. hirsutum*	SCW master regulators: analogous to Arabidopsis MYB46/83 and VND TFs; orchestrate secondary-wall biosynthesis	SCW	–	Expression profiling; homology inference (correlative)	Zhong, Ruiqin, and Zheng-Hua Ye. 2012
GhMYB52-like	*G. hirsutum*	Represses lignin biosynthesis genes in fibers; loss reduces lint index and increases lignin, indicating role in cell-wall composition.	Elongation → SCW	Cell-wall metabolism	CRISPR knockout + phenotyping (exp.)	Yang, Yang et al.2024
GhMYB83-like	*G. hirsutum*	Partner of MYB46 in SCW network; may act in fiber SCW gene activation.	SCW	MYB / SCW network	Expression correlation; functional tests pending (predicted)	Zhong, Ruiqin, and Zheng-Hua Ye. 2012
GhMYBL1	*G. hirsutum*	MYB TF; drives CesA4/7/8, boosts SCW	SCW	Auxin & Ethylene	OE	Wang et al. 2023
GhMYC2	*G. hirsutum*	Basic helix–loop–helix TF in JA signaling; interacts with GhJAZ2. Implicated in fiber initiation regulation through the JA–MYC2 module.	Initiation	JA signaling	Y2H shows GhMYC2–GhJAZ2 interaction; expression enriched around initiation; functional inference from	Hu et al. (2016)

GhMYC3	*G. hirsutum*	bHLH TF in JA signaling; potential partner of GhMYC2; interacts with JAZ proteins in cotton.	Initiation	JA signaling	Y2H shows interaction with GhJAZs; expression profiling suggests involvement; fiber phenotype not directly tested. (interaction evidence)	Yuan et al., 2023
GhNAC TFs (e.g., GhNAC83)	*G. hirsutum*	NAC family TFs regulating SCW genes; some recently shown to bind CesA promoters or modulate wall thickening.	Transition → SCW	NAC / SCW transcriptional network	Expression + ChIP + VIGS (exp./correlative)	Sun, Heng et al. 2018
GhNACs (e.g., GhNAC83)	*G. hirsutum*	NAC TFs regulating SCW genes: bind CesA promoters or modulate wall thickening	SCW	–	Expression + ChIP + VIGS (exp./correlative)	Sun, Heng et al. 2018
GhNCED family (other paralogs)	*G. hirsutum*	Additional NCED isoforms contributing to ABA biosynthesis; stage-specific expression may fine-tune ABA levels across fiber development	Elongation → Transition	ABA biosynthesis	Expression profiling across stages; functional VIGS or overexpression in ovule culture shows influence on ABA level and fiber traits (exp./correlative)	Wu et al., 2018
GhNCED1	*G. hirsutum*	9-cis-epoxycarotenoid dioxygenase; key enzyme in ABA biosynthesis; elevated expression correlates with increased ABA and reduced elongation	Elongation → Transition	ABA biosynthesis	Expression profiling (RNA-seq/qPCR) shows peak late elongation; VIGS in ovules reduces ABA levels and partially rescues elongation (exp./correlative)	Pei et al., 2021

GhNINJA	*G. hirsutum*	NINJA homolog that bridges JAZ repressors to TPL corepressors; inferred to participate in JA-mediated repression of fiber initiation via JAZ complexes.	Initiation	JA signaling	Y2H or co-immunoprecipitation (CoIP) shows GhNINJA interacts with some GhJAZs; expression in ovule epidermis reported. (moderately validated at molecular interaction level)	Wang et al., 2017
GhNPR1	*G. hirsutum*	Nonexpressor of PR genes 1 homolog; master regulator of SA signaling. Likely mediates SA-induced gene expression in cotton; potential cross-talk with fiber stress resilience.	Elongation → Transition	SA perception/signaling	Homology inference; some expression data under pathogen/stress; no direct fiber phenotype studies (predicted/moderately validated)	Zhang, Ying et al.2008
GhOMT1	*G. hirsutum*	O-methyltransferase in flavonoid branch; loss-of-function leads to anthocyanidin over-accumulation and novel fiber colors	Fiber color; SCW	–	Mutant analysis (exp.)	Fan et al.
GhOPR (family)	*G. hirsutum*	12-oxo-phytodienoate reductases reduce OPDA to OPC in JA biosynthesis. Some members show ovule/fiber expression.	Initiation	JA biosynthesis	Expression correlation across initiation; functional knockouts not yet described for fiber. (correlative)	Wang L. et al. (2015)
GhOPR3	*G. hirsutum*	OPR paralog showing expression in ovule/fiber tissues.	Initiation	JA biosynthesis	Expression correlation; no reverse genetics yet. (correlative)	Hu et al., 2018
GhPAG1	*G. hirsutum*	An AP2/EREBP-like TF; modulates BR sensitivity	Elongation	BR regulators	OE & Y2H (exp.)	Yang et al. 2014

GhPAL family	*G. hirsutum*	Phenylalanine ammonia-lyase; contributes to SA via phenylpropanoid pathway. Some paralogs show expression during fiber development, potentially modulating SA pool or related phenolics.	Initiation → Elong.	SA biosynthesis (alternative)	Expression correlation (RNA-seq); functional fiber-specific tests lacking (correlative)	Fan et al., 2012
GhPAS1	*G. hirsutum*	PAS-domain protein; suppressor of GhPAG1, fine-tunes BR output	Elongation	BR regulators	CRISPR (exp.)	Wu et al. 2021
GhPel	*G. hirsutum*	Pectate lyase; degrades de-esterified pectin, enabling fiber elongation	Elongation	–	Fiber-enriched expression; mutation (in Li mutants) leads to poor elongation	Wang, Haihai et al. 2010
GhPEL48_Dt	*G. hirsutum*	Pectate lyase-like: promotes fiber initiation via cell-wall modification, possibly mediated by histone acetylation	Initiation	–	Expression profiling; functional analyses in cotton (fiber initiation)	Zhong, Anlin et al.2024
GhPEL76	*G. hirsutum*	Pectate lyase-like: degrades pectin in primary cell wall, promoting cell-wall loosening during fiber elongation	Elongation	–	Expression profiling; overexpression in Arabidopsis; cotton fiber assays	Sun, Huiru et al. 2020
GhPIN1a / GhPIN1b	*G. hirsutum*	Redundant PIN1 isoforms contributing to polar auxin transport into fiber initials	Initiation	Auxin transport	VIGS double-silence reduces fiber number & length	Wang et al. 2019
GhPIN2	*G. hirsutum*	Auxin efflux carrier family member; potential role in auxin distribution during elongation or transition; expression detected in fibers.	Elongation	Auxin transport	Expression profiling; functional tests pending (correlative)	He, P., Zhao, P., Wang, L. et al. 2017
GhPIN3a	*G. hirsutum*	Auxin efflux carrier; mediates auxin localization for initiation	Initiation	Auxin (IAA)	Ovule-specific RNAi (multiple GhPINs) blocks initiation	Zhang et al. 2017
GhPIN6	*G. hirsutum*	Auxin efflux carrier; may influence auxin distribution during initiation and elongation; expression bias suggests a role in fiber.	Initiation → Elongation	Auxin transport	Expression profiling + homoeolog bias analysis	Mi Zhang et al. 2017
GhPIP2;7 / GhPIP2-2	*G. hirsutum*	Aquaporin: facilitates water influx, turgor during elongation; expression correlates with elongation rate	Elongation	–	Expression profiling; functional assays in ovules (exp./correlative)	Guo, Anhui et al. 2022

GhPOD / GhPRX	*G. hirsutum*	Peroxidases: involved in ROS homeostasis and cell-wall cross-linking during maturation	SCW / Maturation	–	Expression profiling; functional inference (correlative)	Duan, Pengfei et al. 2019
GhPOD2	*G. hirsutum*	Class III peroxidase; expressed in initiation and elongation phases	Initiation, Elongation	–	Accumulates H_2O_2; upregulated in early fiber stages	Guo et al.2007
GhPP2C (e.g., GhABI1, GhABI2, GhHAI2, GhAHG3)	*G. hirsutum*	Protein phosphatase 2C clade; negative regulators of ABA signaling; interact with GhPYLs; likely modulate ABA effects on fiber cells	Initiation → Elongation	ABA signaling	Silencing GhHAI2/GhAHG3/GhABI2 alters ABA responses in stress assays; expression detected in ovules; functional tests in fiber context limited but inferred from stress and expression (moderately validated)	Hamna Shazadee et al.2022
GhPR1 / GhPR2	*G. hirsutum*	Pathogenesis-related proteins induced by SA signaling. While primarily defense-related, their expression in developing bolls/fibers under SA treatment suggests possible roles in cell wall remodeling or stress mitigation.	Transition → Maturation	SA downstream targets	Induced by exogenous SA; expression measured in bolls/fibers; no direct functional analysis on fiber quality (correlative)	Wu, Pan et al. 2023
GhPRE1	*G. hirsutum*	HLH co-activator in PRE/FP/ACE module; enhances elongation when BR high	Elongation	BR regulators	OE & VIGS (exp.)	Wu et al. 2023
GhPRE1 / GhPRE5	*G. hirsutum*	Small HLHs that antagonize FP proteins, derepressing BR/auxin growth module	Elongation	Auxin & BR co-targets	OE lengthens fibers; interacts with ACE1	Wu et al. 2023
GhPSY / GhCRTISO (carotenoid genes)	*G. hirsutum*	In pigment or antioxidant pathways: potential roles in colored fiber or stress resilience, indirectly affecting quality	Fiber color; maturation	–	Expression profiling in colored lines (correlative)	Cai, Caiping et al.2014

GhPSY2D	*G. hirsutum*	Phytoene synthase; enhances carotenoid precursors for ABA; up-regulation raises ABA in fibers, promoting SCW initiation	Transition → SCW	ABA biosynthesis (indirect)	OE in fibers increases carotenoids and ABA, advancing SCW onset (exp.)	Chuannan Wang et al.2025
GhPYL family (e.g., GhPYL8d2, GhPYL9, GhPYR1-3A)	*G. hirsutum*	PYR/PYL/RCAR ABA receptors; perceive ABA to inhibit PP2C phosphatases, initiating ABA signaling; expression detected in ovules/fibers	Initiation → Elongation; Transition	ABA perception	Genome-wide identification of ~40 GhPYLs; expression profiling shows several preferentially in ovule/fiber; OE in Arabidopsis/cotton shows ABA-responsive phenotypes (stress-focused); direct fiber phenotypes inferred from ABA treatment effects (correlative/partial exp.)	Liu et al., 2023
GhRacA	*G. hirsutum*	Rho-type GTPase; promotes cell expansion during elongation	Elongation	–	Fiber-preferential expression; candidate from SSH libraries	LiI, Xian-Bi et al. 2005
GhRacB	*G. hirsutum*	Rho-type GTPase; active in secondary wall deposition	Secondary CW	–	Expressed in SCW stage; may regulate late expansion	LiI, Xian-Bi et al. 2005
GhRBOH	*G. hirsutum*	NADPH oxidase: generates ROS signals in fiber cells; affects cell-wall loosening and signaling	Elongation / Initiation	–	Expression and inhibitor studies (correlative)	Wang, Wei et al. 2020
GhRDL1	*G. hirsutum*	Cell-wall remodelling; prevents "ribbon-like'' weak fibers	Elongation	—	Mutant & RNAi	Wang et al. 2004; Xu et al. 2013
GhREV_D5	*G. hirsutum*	HD-ZIP III TF; similar to GhHB14_D10 in SCW regulation; affects fiber length and wall properties.	Elongation → SCW	HD-ZIP III / SCW	VIGS silencing + phenotyping (exp.)	Li, Shuaijie et al. 2024

GhRLKs (other families)	*G. hirsutum*	Receptor-like kinases (e.g., LRR-RLKs, lectin RLKs) found in transcriptomic screens with fiber-preferential expression; may mediate external signals.	Initiation → Elongation	RLK signaling	Expression profiling + co-expression network inference (correlative)	Yuan et al., 2018
GhROP6	*G. hirsutum*	ROP GTPase restricts GhPIN3a at PM, sharpening auxin peaks; OE lines show longer fibers	Pre-initiation & Elongation	Auxin-modulated signalling	OE & dominant-negative mutants; live-cell PIN imaging	Xi et al. 2023
GhROPGEF5	*G. hirsutum*	ROP-GEF; modulates elongation & SCW deposition	Elong. → Transition	—	CRISPR + OE	Wang et al. 2024
GhSAUR50-1 /-2	*G. arboreum*	SAURs that stimulate H^+-ATPase, enhancing turgor-driven expansion	Early–Mid Elong.	Auxin rapid-response genes	OE in Arabidopsis and cotton increases fiber length	Li et al. 2022
GhSK13	*G. hirsutum*	GSK3/SHAGGY kinase integrating BR and ethylene signalling	Initiation + Elong.	BR ↔ Ethylene	OE & kinase assay (exp.)	Wang et al. 2020
GhSLR1 (DELLA)	*G. hirsutum*	DELLA repressor; inhibits GA-mediated elongation	Elongation	GA	RNAi/overexpression studies (DELLA mutant)	Shan et al. 2014; Sun et al. 2024
GhSMO2-2	*G. hirsutum*	Sterol C-4 methyl oxidase; BES1 target, promotes BR biosynthesis	Elongation	BR targets	OE & ChIP (exp.)	F. Liu et al. 2023
GhSMT2–1	*G. hirsutum*	Sterol methyltransferase: alters phytosterol levels; overexpression increases sitosterol, reduces campesterol, affecting elongation and SCW thickening	Elongation / SCW	–	OE in cotton (exp.)	Luo, Ming et al.2008
GhSMXL homologs (other D53/SMXL family members)	*G. hirsutum*	Additional SMXL paralogs potentially functioning as SL repressors in fiber development	Elongation	SL signaling	Expression profiling across fiber stages; functional roles inferred by homology to GhSMXL7/8; specific studies pending (correlative)	Sun et al. 2024
GhSMXL7	*G. hirsutum*	SL repressor; blocks GA/Auxin & HOX3	Elongation	SL / GA / Auxin	CRISPR + OE	Sun et al. 2024

GhSMXL7 / GhSMXL8	*G. hirsutum*	Strigolactone repressors that directly bind ARF promoters, blocking auxin output until SL degrades them	Elongation	Auxin–SL crosstalk repressors	CRISPR double KO: longer fibers; ChIP-qPCR on ARF promoters	Sun et al. 2024
GhSMXL8	*G. hirsutum*	Redundant with GhSMXL7	Elongation	SL / GA / Auxin	CRISPR + OE	Sun et al. 2024
GhSnRK2 (e.g., GhSnRK2.6 homologs)	*G. hirsutum*	SNF1-related protein kinase 2; activated upon PP2C inhibition; phosphorylates downstream TFs like AREB/ABF; potential role in mediating ABA responses in fibers	Elongation → Transition	ABA signaling	Homologs identified, expression upregulated by ABA in ovule culture; direct fiber phenotype data lacking—predicted role from expression (correlative)	Liu, Zhao et al. 2017
GhSUS2	*G. hirsutum*	Sucrose synthase; target of GhCPK84/93 phosphorylation; altered activity under ABA shifts carbohydrate partitioning, hindering elongation	Late Elongation	ABA signaling	Phospho-site mutation studies in vitro; expression changes under ABA; phenotypic correlation in fiber elongation assays (exp./correlative)	Wang, Yao et al. 2023
GhSusA1 (Sus1)	*G. hirsutum*	Sucrose synthase; supplies UDP-glucose for cellulose, promotes elongation	Elongation	–	RNAi and OE studies: suppression reduces fiber length/quality, OE increases length and cell-wall thickness	Jiang, Yanjie et al. 2012
GhTCP14	*G. hirsutum*	TCP family TF; promotes both initiation and elongation	Initiation, Elongation	–	Detected in early ovules and fibers	Wang, Miao-Ying et al. 2013
GhTCP15	*G. hirsutum*	Interacts in the GhMYB4 module; contributes to repressing elongation when promoted by GhMYB4.	Transition	TCP / elongation–SCW	Interaction assays + expression (exp.)	Qiao, Lu et al. 2025
GhTER (trans-2-enoyl-CoA reductase)	*G. hirsutum*	Fatty acid elongation enzyme: affects very-long-chain fatty acid composition; implicated in fiber elongation	Elongation	–	Characterization in J. Exp. Bot. (exp.)	Song, Wen-Qiang et al. 2009

GhTGA (e.g., GhTGA2)	*G. hirsutum*	bZIP TFs interacting with NPR1 to activate PR genes in SA signaling. Cotton homologs expressed under stress; potential indirect effects on fiber cell wall via defense-related pathways.	Elongation → Maturation	SA signaling	Expression profiling under SA or stress treatments; direct fiber development tests lacking (correlative)	Zhong, Xionghui et al.2015
GhTRY	*G. hirsutum*	TRIPTYCHON-like MYB; negative regulator of initiation	Initiation	–	Expressed in ovule epidermis; represses initiation	Wang, Gaskin et al. 2013
GhTTG1	*G. hirsutum*	WD40 repeat protein; positive in initiation, part of MYB–bHLH–WD40 complex	Initiation	–	Complementation of Arabidopsis ttg1 mutant restores trichomes	Tian, Yue et al.2020
GhTTG2	*G. hirsutum*	WD40 repeat protein; positive in initiation	Initiation	–	Co-expressed with GhMYB2; functional homologs of GL1 pathway	Wan, Qun et al. 2014
GhTTG3	*G. hirsutum*	WD40 repeat protein; positive in initiation	Initiation	–	Rescues trichome development in ttg1 mutants when overexpressed	Tian, Yue, and Tianzhen Zhang. 2021
GhTTG4	*G. hirsutum*	WD40 repeat protein; positive in initiation	Initiation	–	Expressed in early ovule epidermis; function inferred from WD40 family	Tian, Yue et al.2020
GhTUB / GhTUA (e.g., GhTUB1, GhTUA9)	*G. hirsutum*	Tubulin α/β subunits: essential for microtubule assembly; influence cell elongation and fiber strength	Elongation; SCW	–	Expression profiling; overexpression/VIGS (exp.)	GhTUB1 characterization; GhTUA9 OE in yeast and fiber studies
GhTZF2	*G. hirsutum*	Tandem zinc finger protein; interacts with GhMORF8; regulates fiber cell development, likely via RNA metabolism or stress responses.	Initiation → Elongation	RNA-binding / post-transcriptiona l	Interaction assays + expression profiling (exp.)	Li, Y et al. 2023

GhUGT74F1	*G. hirsutum*	UDP-glucosyltransferase that conjugates SA to SA-glucoside; modulates active SA pool. Cotton homologs may affect dynamic SA availability in developing fibers.	Elongation	SA conjugation	Expression profiling indicates paralog expression in fibers; functional tests pending (correlative)	Chen, Bowen et al. 2024
GhVND homologs	*G. hirsutum*	NAC TFs analogous to VND (vessel-NAC) in Arabidopsis; potential regulators of SCW program in fibers.	SCW	NAC / SCW pathway	Expression profiling; homology inference (correlative)	Sun, H., Hu, M., Li, J. et al. 2018
GhWBC1	*G. hirsutum*	ABC transporter highly expressed in rapidly elongating fibers (peaks 5–9 DPA); correlates with fiber elongation	Elongation	–	Expression profiling; transgenic in Arabidopsis (short silique phenotype)	Zhu, Yong-Qing et al.2003
GhWDL3	*G. hirsutum*	WAVE-DAMPENED-LIKE protein: modulates cortical microtubules; overexpression alters fiber morphology; silencing affects elongation	Elongation	–	Expression & VIGS in cotton/Arabidopsis (exp.)	(e.g., WDL3 functional assays)
GhWER	*G. hirsutum*	WER-type MYB; triggers ethylene burst for initiation	Initiation	Ethylene	CRISPR	Zhao et al. 2024
GhWRKY family	*G. hirsutum*	WRKY TFs (e.g., GhWRKY70) involved in SA-mediated defense. Some members expressed during fiber development; may influence cross-talk between defense and cell-wall processes.	Elongation → Transition	SA signaling crosstalk	Expression profiling and some overexpression in stress contexts; fiber-specific roles inferred (correlative)	Dou, Lingling et al. 2014
GhWRKYs (specific members)	*G. hirsutum*	Beyond defense roles, some WRKY TFs modulate ROS homeostasis or cell-wall-related genes in maturing fibers.	Transition → Maturation	SA/ROS crosstalk	Expression profiling + overexpression in stress context (correlative)	Dou, Lingling et al. 2014
GhXB38D	*G. hirsutum*	E3 ligase; ubiquitinates ACS4/ACO1 to cap ethylene	Elongation	Ethylene	OE + KO	Song et al. 2023
GhXTH1	*G. hirsutum*	Xyloglucan endotrans-glycosylase; wall loosening (BZR1 target)	Rapid Elong.	BR	OE + ChIP	Sun et al. 2005
GhZFP8	*G. hirsutum*	GA-released zinc-finger; activates SDCP1→PIF3	Elongation	GA	OE + KO	He et al. 2024

GrCesA	*G. raimondii*	Cellulose synthase genes: baseline SCW biosynthesis; serves as diploid reference for comparative studies	SCW	–	Genome annotation & expression inference (correlative)	Kim et al., 2019
Li2 locus	*G. hirsutum*	Dominant ta-siRNA mutation; extremely short fibers	Initiation / Elong.	—	Natural mutant	Naoumkina et al. 2014; Patel et al., 2020
PRE1	*G. arboreum*	PRE1 homolog: may modulate cell elongation signaling; expressed during elongation though less studied in diploid	Elongation	–	Expression data from initiation/elongation transcriptomes (correlative)	Zhao et al., 2018; Liu et al., 2015
