# Supplementary material for: Genetic basis and role of exotic accessions in cultivated cotton fiber quality improvement
Source: Theor Appl Genet. 2025 Sep 27;138(10):260. doi: 10.1007/s00122-025-05043-2 (PMC12476451; doi:10.1007/s00122-025-05043-2)
Supplement: Supplementary file 2 — Supplementary file2 (PDF 158 kb) [file 122_2025_5043_MOESM2_ESM.pdf]

Supplemental Table 1. Landmark genomic resources, molecular breakthroughs, and breeding innovations that have driven cotton fiber-quality research and improvement (1926 – 2025).

Year	Notable Research	What was achieved	Impact on fiberfiber science	Sources
1926	NMSU *Acala-1517* public-breeding programme launched	First systematic long-staple selection in the U.S. Southwest	Alleles from Acala-1517 still underpin ≈45 % of modern Upland cultivars; model for length/strength	(Staten, 1973)
1978	USDA rolls out High-Volume Instrument (HVI) classing	Automated, high-throughput measurement of length, strength, micronaire & colour	Replaced subjective hand-classing, enabling nationwide genetic studies of fiber quality	(Brenni, 2013)
1994	Reinisch *et al.* 705-locus RFLP linkage map	First high-density cotton map (4 675 cM) spanning both sub-genomes	Opened the door to QTL mapping for length, strength, fineness & uniformity	(Reinisch et al., 1994)
2000	Ulloa & Meredith first intraspecific QTLs for fiber traits	Mapped length & strength loci in a *Gh* × *Gh* population	Proved elite germplasm still harbours exploitable diversity	(Ulloa & Meredith, 2000)
2007	meta-analysis of >1 100 fiber QTL	Integrated scattered studies; revealed asymmetric At/Dt “hot-spots”	Guided map-based cloning & MAS targets still used today	(Rong et al., 2007)
2010	Walford *et al.* GhMYB25-like cloned	First master TF shown essential for lint initiation vs. fuzz	Established the genetic entry-point for engineering fiber cells	(S.-A. Walford et al., 2011)

2012	Jiang *et al.* GhSusA1 over-expression	Sucrose-synthase increased fiber length (+6 %) & strength (+9 %)	Proof-of-concept metabolic boost for carbon allocation	(Jiang et al., 2012)
2012	Paterson *et al.* draft genome of *diploid G. raimondii*	567 Mb, 40 976 genes; 73 % anchored	First cotton reference foundation for all later -omics	(Andrew H Paterson et al., 2012)
2014	Li *et al.* draft genome of *diploid G. arboreum*	1.65 Gb, 41 330 genes	Completed A-genome partner; enabled ancestral reconstructions & homoeolog analysis	(Li et al., 2014)
2015	Li *et al.* draft allotetraploid *G. hirsutum* TM-1	2.3 Gb; 66 434 genes across At/Dt	First look at the production genome that supplies >90 % of world fiber	(Li et al., 2015)
2015	Liu *et al.* draft genome of *G. barbadense*	2.57 Gb extra-long-staple (ELS) genome	Illuminated genetic basis of superior length & fineness in Pima cotton	(Liu et al., 2015)
2015	Bajwa *et al.* GhEXPA8 expansin transgenics	Enhanced fiber length (+17–20 %) & lowered micronaire	Demonstrated cell-wall loosening as a lever to tune both length & fineness	(Bajwa et al., 2015)
2016	Zhang *et al.* multi-omics CHH-methylome maps	Discovered CHH surge & RdDM shutdown during elongation	Linked epigenetic re-programming to single-cell growth dynamics	(Wang et al., 2016)

2018	Zhang & Li *et al.* PacBio + Hi-C reference-grade TM-1 & 3-79	Contig N50 > 20 Mb; centromeres resolved	Set gold-standard assemblies; accelerated marker assays & GWAS	(M. Wang et al., 2019)
2020	Huang *et al.* updated TM-1 v2 & new *G. herbaceum* genome	TM-1 contig N50 5 Mb; 95.7 % of *G. herbaceum* anchored	Gave breeders chromosome-level diploid & tetraploid references	(Huang et al., 2020)
2021	Wang *et al.* 1 961-accession cotton pan-genome	Added 32 569 non-reference genes & 162 domestication loci	Delivered presence/absence markers & rare alleles for length/strength	(Li et al., 2021)
2022	Wen *et al.* cellulose-synthase super-complex	Revealed 36-mer GhCesA4/7/8 complex unique to secondary walls	Explains cotton's exceptional cellulose deposition & tensile strength	(Wen et al., 2022)
2022	Pei *et al.* Hi-C 3D-genome atlas of fiber development	10 571 TAD-like domains; stage-specific loops & subgenome bias	Connected chromatin architecture to homoeolog expression driving quality traits	(Pei et al., 2022)
2023	Hu *et al.* GhROD1 down-regulation	Lipid-pathway tweak delayed wall thickening, producing finer fibers	First demonstration that linoleic-acid signalling modulates fineness	(Ding et al., 2024)

2024	Huang *et al.* telomere-to-telomere (T2T) assembly of *G. raimondii*	Closed all 13 centromeres & 25 telomeres	Corrected legacy mis-assemblies; new insights into centromere evolution	(Huang et al., 2024)
2025	Hu *et al.* T2T assembly of *G. hirsutum* cv. Zhongmian 113	Resolved 26 centromeres & 52 telomeres; identified short-season inversion	Provides a precision reference for climate-adapted breeding	(Hu et al., 2025)
2025	Meng *et al.* graph pan-genome of *G. barbadense*	12 new HiFi genomes + 17 public assemblies; SV-GWAS for ELS traits	Captures structural-variant diversity for precision improvement of Pima fiber	(Meng et al., 2025)
2025	Sun *et al.* spatiotemporal single-cell + metabolome atlas	Mapped 51 k protoplasts & metabolites (e.g., DOX2, KCS19) through initiation	Roadmap for cell-type-targeted engineering of length, fineness & wall composition	(Sun et al., 2025)
2025	Xie *et al.* duplicated GhMML3 genes dissected	Showed dose-dependent control of lint + fuzz initiation by GhMML3_A12/D12	New CRISPR targets for fine-tuning fiber density & staple length	(Chen et al., 2025)
